# Genotyping-by-sequencing reveals range expansion of *Adonis vernalis* (Ranunculaceae) from Southeastern Europe into the zonal Euro-Siberian steppe

**DOI:** 10.1038/s41598-022-23542-w

**Published:** 2022-11-09

**Authors:** Anna Seidl, Karin Tremetsberger, Simon Pfanzelt, Lisa Lindhuber, Matthias Kropf, Barbara Neuffer, Frank R. Blattner, Gergely Király, Sergey V. Smirnov, Nikolai Friesen, Alexander I. Shmakov, Kristina Plenk, Oyuntsetseg Batlai, Herbert Hurka, Karl-Georg Bernhardt

**Affiliations:** 1 Institute of Botany, Department of Integrative Biology and Biodiversity Research, University of Natural Resources and Life Sciences, Vienna, Gregor-Mendel-Straße 33, 1180 Vienna, Austria; 2 Experimental Taxonomy, Leibniz Institute of Plant Genetics and Crop Plant Research, 06466 Gatersleben, Germany; 3 Institute for Integrative Nature Conservation Research, Department of Integrative Biology and Biodiversity Research, University of Natural Resources and Life Sciences, Vienna, 1180 Vienna, Austria; 4 School of Biology/Chemistry, Osnabrück University, 49076 Osnabrück, Germany; 5 Faculty of Forestry, University of Sopron, 9400 Sopron, Hungary; 6 South-Siberian Botanical Garden, Altai State University, 656049 Barnaul, Russia; 7 Botanical Garden of the Osnabrück University, 49076 Osnabrück, Germany; 8 Department of Biology, School of Arts and Science, National University of Mongolia, 14201 Ulaanbaatar, Mongolia; 9 Present Address: Botanical Garden München-Nymphenburg, 80638 Munich, Germany

**Keywords:** Biogeography, Phylogenetics

## Abstract

The Euro-Siberian steppe flora consists of warm- and cold-adapted species, which may have responded differently to Pleistocene glacials and interglacials. Genotyping-by-sequencing individuals from across the distribution range of the pheasant’s eye (*Adonis vernalis*), we aimed to gain insight into steppe florogenesis based on the species’ evolutionary history. Although the primary area of origin of the species group comprising *A. vernalis*, *A. villosa* and *A. volgensis* is in Asia, our results indicate that recent populations of *A. vernalis* are not of Asian origin but evolved in the southern part of Europe during the Pleistocene, with Spanish populations clearly genetically distinct from the Southeastern European populations. We inferred that *A. vernalis* migrated eastwards from the sub-Mediterranean forest-steppes of Southeastern Europe into the continental forest-steppe zone. Eastern European populations had the highest private allelic richness, indicating long-term large population sizes in this region. As a thermophilic species, *A. vernalis* seems unlikely to have survived in the cold deserts of the Last Glacial Maximum in Western Siberia, so this region was likely (re)colonized postglacially. Overall, our results reinforce the importance of identifying the area of origin and the corresponding ecological requirements of steppe plants in order to understand the composition of today’s steppe flora.

## Introduction

**Figure 1:**
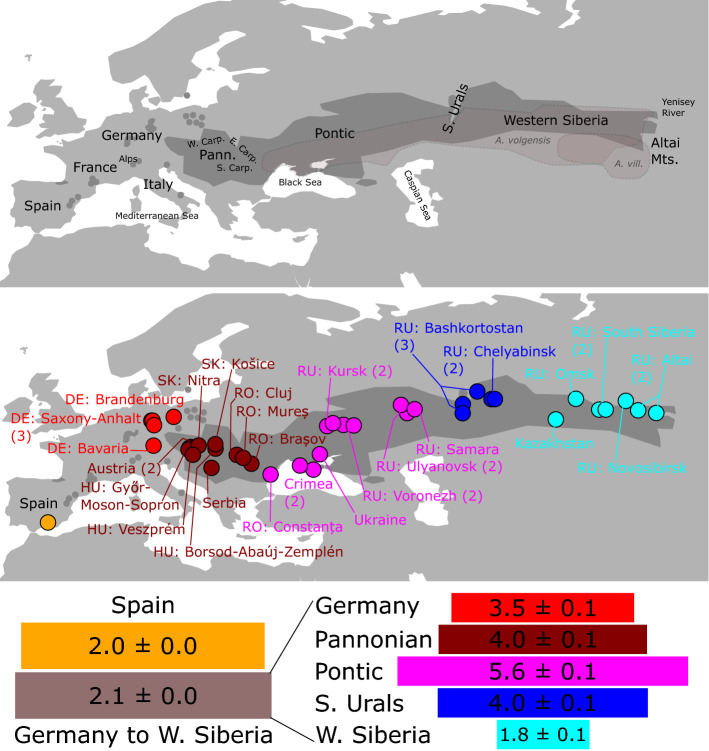
Top: Map of Europe and Middle Asia showing the approximate distribution ranges of *Adonis vernalis* (dark grey), *A. villosa* and *A. volgensis*^21,22^ and important geographical designations used in this study (Carp.—Carpathians; Pann.—Pannonian). Following standard biogeographical literature, we use the term Middle Asia to refer to the region between the Ural Mountains and the Altai Mountains (corresponding to Western Siberia), and the term Central Asia to refer to the region east of the Altai Mountains^1^. Middle: Sampled populations of *A. vernalis* in the Euro-Siberian region. Populations are divided into colour-coded geographic groups (Supplementary Table S4 online). Two-letter abbreviations are ISO alpha-2 country codes. The numbers in parentheses indicate the number of populations (if more than one). Bottom: Private allelic richness (pAr) in two comparisons, left Spain versus all other populations (value = pAr × 10), right geographic groups from Germany to Western Siberia (excluding the Spanish population; value = pAr × 100). Values are the mean ± standard error over 8,539 unlinked SNPs calculated from GBS data. The map was created in R v4.2.1 (https://www.R-project.org/) with the package rworldmap v1.3–6 (https://rdocumentation.org/packages/rworldmap/versions/1.3-6) and edited in Inkscape v0.92 (https://inkscape.org/).

The Eurasian steppes form the largest contiguous temperate grassland region on Earth. They extend from the Mediterranean Basin towards China, being more scattered towards the western and eastern peripheries^1^. The steppe areas display large-scale temperature and precipitation gradients and are divided into lowlands and mountain ranges, resulting in zonation by longitude, latitude, and altitude. In this study we are concerned with the western part of the Eurasian steppes, the Euro-Siberian steppes, which formed around the Altai Mountains in Central Asia (Fig. 1) and extended successively westward^2^. From the Altai Mountains to Central Europe, continentality decreases and precipitation increases. The zonal Euro-Siberian steppes comprise the Middle Asian chorological-climatic subtype between the Altai Mountains and the Ural Mountains (corresponding to southern Western Siberia in Russia and the north of Kazakhstan) and the European chorological-climatic subtype, which reaches from the Ural Mountains to the area northwest of the Black Sea^1^. The European subtype corresponds to the Pontic steppes of Eastern Europe in biogeographic literature^1^. The characteristic zonal Euro-Siberian steppe vegetation extends throughout Eastern Europe and Middle Asia (southern Western Siberia) and consists of semi-dry grasslands of the Festuco-Brometea class, which adjoin the moister meadow steppes of the forest-steppe zone to the south^1,3^. The Pannonian region to the west is home to forest-steppe. Scattered patches of semi-natural azonal or extrazonal steppe-like grasslands resembling the climate-driven zonal steppes are found in forest climate in Central Europe as a result of human land use and/or on sites with dry edaphic conditions, for example on rocky or sandy terrain^1,2^.

It is assumed that archetypes of steppe characterised by a more savannah-like habit and the occurrence of nowadays extinct (mega) herbivores such as *Hipparion* species spread from Asia to Europe in the Miocene under the then relatively warmer climate^2^. At the beginning of the Quaternary, in the Lower Early Pleistocene (2.6–1.8 Ma), global cooling, strong aridification, and increased seasonality of the climate led to a further opening of the landscape and the spread of more modern types of steppes^2,4^, a development that intensified further during the transition from the Early to the Middle Pleistocene (0.9 Ma)^5,6^. It is likely that the Pleistocene glacial and interglacial cycles resulted in alternating advances and retreats of warm- and cold-adapted species, respectively. During the cold periods, plant communities existed that did not or do not occur in this composition in the warm periods either before or after^7^, so that the repeated range shifts under alternating favourable growing conditions probably led to a mixing of species from different phytogeographic regions (Euro-Siberian, Irano-Turanian, and Mediterranean^1,2,8^).

Molecular phylogenetic studies have confirmed that many steppe plants belong to genera or species groups within genera that originated in the Irano-Turanian floristic region such as for example the genera *Astragalus*, *Camelina*, *Capsella*, *Clausia*, *Dontostemon*, *Krascheninnikovia* and *Sisymbrium* as well as various species groups within *Allium*^8–15^. At a lower taxonomic level, namely the species level, various studies place the beginning of the spread and diversification of the present-day steppe species between the Early Pleistocene and the Holocene, and the areas of origin of these species from the Caucasus region at the border between Europe and Asia to the Altai Mountains region in Asia, for example for *Astragalus onobrychis* L., *Capsella orientalis* Klokov, *Clausia aprica* (Stephan ex Willd.) Korn.-Trotzky and *Krascheninnikovia ceratoides* (L.) Gueldenst^11,12,14,16,17^. Other species descended from Euro-Siberian/Circumboreal groups. For example, *Schivereckia podolica* Andrz. & Besser ex DC. may have immigrated into the steppe from an ancient arctic/subarctic vegetation belt via the Ural Mountains, which mark the boundary between Asia and Europe^18^. However, the areas of origin and evolutionary histories of many steppe plant species remain unknown because there are few range-wide phylogeographic studies due to the sheer size of the Eurasian steppe area. The response patterns of these species to Pleistocene climate fluctuations are also still poorly known.

From a chorological point of view, the submeridional and thermophilic pheasant’s eye (*Adonis vernalis* L.) is representative for the Euro-Siberian steppe flora and therefore a suitable model to study the history of the semiarid Euro-Siberian steppes and forest-steppes. For example, the species is listed in biogeographical literature as a “Pontic geoelement^19^” or in floristic works as growing “in dry and steppe grasslands (meadow steppes), also in pine forests^20^”. Occurring from Spain in the west to near the Yenisey River in Siberia in the east, its distribution is continuous in the east, but disjunct in the west (Fig. 1)^21,22^. In Eastern (and Southeastern) Europe and Western Siberia, the species grows in steppes, in the transition zones between forest and steppe, and in open birch forests. The plants are found on chernozem soil with high carbonate content, rocky slopes, and secondarily on cattle resting places^22^. In Central Europe, the species is found in extrazonal calcareous dry-warm grasslands in the deciduous forest zone^3^. From a phytosociological perspective, *A. vernalis* is listed as an “indicator species for steppe grasslands^23^”. While it is not “diagnostic” (in the statistical sense) for the order Brachypodietalia (= meadow steppes), it is diagnostic for the alliance “Cirsio-Brachypodion pinnati” within this order^3^ (as well as for the entire class Festuco-Brometea^23^), i.e., it can be called a typical “meadow steppe species”. The “meadow steppes” are those open grasslands that occur in the “forest-steppes” interspersed with (groups of) trees, which *A. vernalis* underlines by also colonizing light e.g., pine forests. The pheasant’s eye is an early-blooming, poisonous perennial with a vigorous sympodial rhizome that produces a dense clump of shoots that (if flowering) terminate in a showy yellow flower. It is pollinated by insects^20,24,25^. The nutritious elaiosomes of the nutlets attract ants, which then disperse the seeds (myrmecochory)^20,24^.

The aim of this study was to determine the evolutionary history of the populations of the pheasant’s eye growing in the Euro-Siberian steppe and to interpret their history in relation to the formation of the Euro-Siberian steppes. We were interested in when, where, and in connection with which Earth history events recent populations of *Adonis vernalis* originated and which factors affected their diversification and spread into the present range of the species. We hypothesize that thermophilic species adapted to semiarid climates, such as *A. vernalis*, responded in different or even opposite ways to Quaternary climate changes than eurythermal species adapted to arid climates or cold-adapted species and that they may not have benefited from glacial cold phases to the same extent as these other species. For example, the eurythermal *Krascheninnikovia ceratoides* is thought to have expanded its range as part of the cold steppe vegetation that spread during the glacial periods^12^. Thermophilic steppe species, by contrast, may have had to retreat to glacial refugia with a warmer (local) climate. In this context, we were also interested in where any refugia of *A. vernalis* were located during the cold phases of the Pleistocene. To achieve this aim, we genotyped individuals in populations from across the range of *A. vernalis*, as well as individuals from closely related species, using genotyping-by-sequencing (GBS) to determine the geographic origin of recent populations of the pheasant’s eye and phylogeographic patterns within the species. The phylogeographic approach was complemented by Sanger sequencing of chloroplast and internal transcribed spacer (ITS) sequences in combination with a secondary calibration approach, which we used to construct a dated phylogeny of the tribe Adonideae to determine the timing of diversification of recent populations of *A. vernalis*.

## Results

### Data retrieved by genotyping-by-sequencing (GBS) and genome size

To investigate genetic relationships at the population level, we applied GBS to 184 individuals of *Adonis vernalis*, five individuals of *A. volgensis* Steven ex DC., and one individual of *A. turkestanica* (Korsh.) Adolf. The ipyrad assembly of the ingroup (*A. vernalis*) and the outgroup (*A. volgensis* and *A. turkestanica*) retained 229,597 single nucleotide polymorphisms (SNPs; out of 4,384,129 characters) across 43,917 loci, of which 39,024 were unlinked SNPs and 23,242 were informative and unlinked SNPs. *Adonis vernalis* samples had on average 23,562 SNPs (with a range of 11,879 to 31,121 SNPs) and outgroup samples had on average 18,613 SNPs (with a range of 15,011 to 21,102 SNPs). The ingroup alignment (*A. vernalis* only) contained 160,130 SNPs (out of 4,399,929 characters) across 44,124 loci, of which 37,152 were unlinked SNPs. In this alignment, the samples had on average 23,903 SNPs, ranging from 12,032 to 31,518 SNPs. The two replicates of the same individual of the *A. vernalis* population from Lower Austria had 98.3% identical SNPs, and the two replicates of the Russian Kalmanka population of *A. vernalis* had 97.6% identical SNPs. Among all other individuals, only five pairs of individuals (which always came from the same population) had a higher similarity (of 99.5%, 99.6% or 99.7%) and might represent ramets of the same plant.

The number of SNPs can be related to genome size and ploidy. *Adonis vernalis* was reported as uniformly diploid (2*n* = 16) with a genome size of approximately 18.9–19.5 pg (2C) in the eastern part of its range in Siberia^26^. Two individuals from Austria measured by us (each from a different locality) had comparable values of approximately 19.3 and 19.5 pg, and one individual (from still another locality in Austria) had a somewhat higher value of approximately 20.1 pg. The values are not so accurate because leaf material dried with silica gel was used. Estimates of ploidy based on GBS data using nQuire and ploidyNGS indicated a diploid genome for all individuals except one from a population from South Siberia (Neudachino), which was most likely triploid according to nQuire, while its ploidy remained unclear using ploidyNGS.

### Population structure based on GBS data

**Figure 2:**
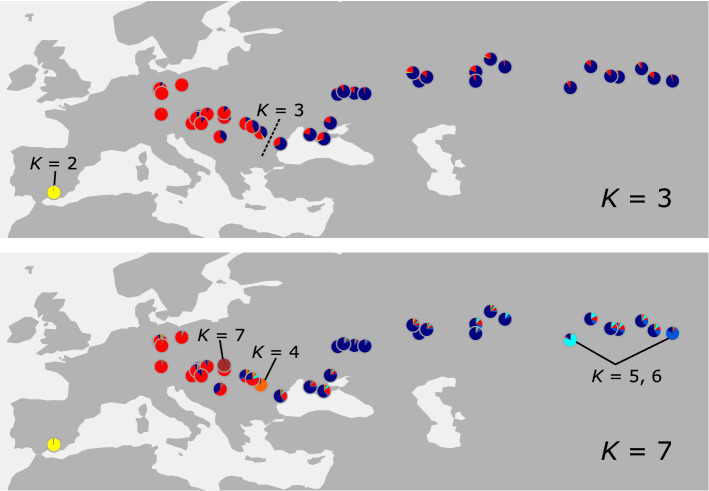
Pie charts (one for each population) created with LEA using the GBS data (ingroup dataset of 37,152 unlinked SNPs with a maximum of 90% missing data per locus) showing the proportions of genetic groups within populations. The optimal number of groups (*K*) according to the minimal cross-entropy criterion is seven.

**Figure 3:**
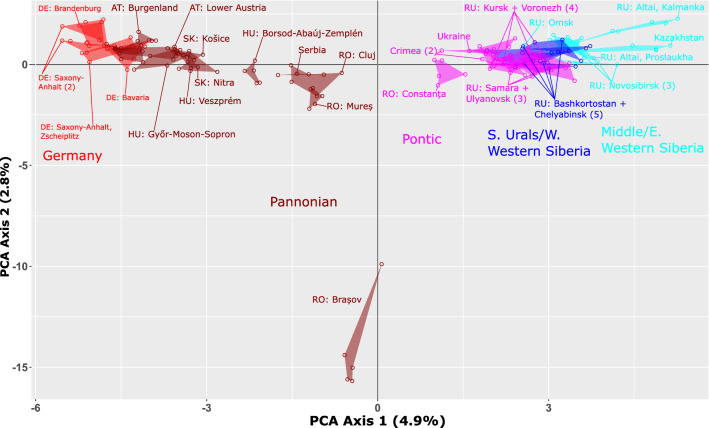
Principal Component Analysis of GBS data of the eastern lineage of *Adonis vernalis* (ingroup dataset without the Spanish population; 8,015 unlinked SNPs with a maximum of 10% missing data per locus). The colour-coding refers to the geographic groups defined in Fig. 1. Two-letter abbreviations are ISO alpha-2 country codes. Overlapping populations (mainly from the Southern Urals and Western Siberia) are not all labelled at the population level. The numbers in parentheses indicate the number of populations (if more than one).

To assess the population genetic structure of *Adonis vernalis*, we employed Bayesian admixture analysis and Principal Components Analysis (PCA). Admixture analysis with LEA found a most likely number of ancestral populations (*K*) of seven according to the cross-entropy value (Fig. 2, Supplementary Fig. S1 online). At *K* = 2, the Spanish population formed its own cluster. The remaining populations were divided into a western and an eastern cluster, with some admixture between them (*K* = 3). The border between the two clusters is along the eastern Carpathians and corresponds to the border between the Pannonian and Pontic regions. Further independent clusters were formed progressively by individuals of the Brașov population in Romania (*K* = 4), the Kazakh population and the Kalmanka population in the Russian Altai region (*K* = 5 and 6), and the Košice population in Slovakia (*K* = 7). In the PCA (Fig. 3; without the Spanish population), the Romanian Brașov population was clearly separated from all other populations, which were arranged along a west–east gradient: the German populations were adjacent to populations from Austria, the Romanian Cluj and Mureș populations together with the Serbian population were placed between the populations west and east of them, and the Pontic populations overlapped with the Western Siberian populations.

### Origin and spread of *Adonis vernalis*

Molecular dating allowed an estimate of the time of origin of *Adonis vernalis*. We chose a secondary calibration approach using node ages reported for the family Ranunculaceae^27^. Before that, we reconstructed the phylogeny of the tribe Adonideae based on maximum-parsimony (MP) and maximum-likelihood (ML) analyses of three chloroplast markers and, separately, the internal transcribed spacer (ITS). The markers showed extensive agreement in the relationships, although there were also incongruencies (Supplementary Fig. S2 online). For example, *A. villosa* Ledeb. was the sister of *A. volgensis* in the chloroplast tree, but the sister of *A. vernalis* in the ITS tree. However, the three species *A. vernalis*, *A. villosa* and *A. volgensis* were resolved as a clade in the chloroplast tree (with 71% and 73% bootstrap (BS) support in the MP and ML analysis, respectively) and in the ITS tree (with 93% and 96% BS support in the MP and ML analysis, respectively). Molecular dating rested on the secondary calibration of three nodes, two of which were much younger than the third node corresponding to the tribe Adonideae (Supplementary Fig. S3 online). In the molecular dating analyses, we tried what the effect would be of choosing different values of sigma for the normal priors of the three calibration nodes. With a sigma of 1.0 for all three calibration nodes, the estimated age of the tribe Adonideae was very similar to the mean of its normal prior, which was set to 25.5 Ma, but the 95% highest posterior density (HPD) interval was too small compared to the reported interval^27^ (Supplementary Fig. S3 online, Supplementary Table S1 online). With a sigma of 5.0 for the tribe Adonideae, which was chosen to better reflect its reported range of 12.7–43.2 Ma^27^ (and all other settings remaining the same), the estimated age of the tribe Adonideae (and equally of all younger nodes in the genus *Adonis*) was shifted. We therefore relied on the more accurate median age estimates of the analyses with a sigma of 1.0 for all three calibration nodes and on the 95% HPD intervals of the analyses with a sigma of 5.0 for the tribe Adonideae for the discussion of the results. According to this, the analyses revealed a crown group age of *A. vernalis* of 0.5 Ma (95% HPD interval = 0.0–1.5 Ma) based on the ITS sequences and also of 0.5 Ma (95% HPD interval = 0.1–1.3 Ma) based on the chloroplast sequences (Supplementary Fig. S3 online, Supplementary Table S1 online). The age of the stem group of *A. vernalis* was estimated to be 1.5 Ma (95% HPD interval = 0.4–3.3 Ma) based on the ITS sequences and 1.6 Ma (95% HPD interval = 0.8–2.5 Ma) based on the chloroplast sequences. Given the extremely low variation in the chloroplast and ITS sequences of *A. vernalis* (see Supplementary Fig. S2 online) and the lack of transmission of the full range of uncertainty through the secondary calibration approach, age estimates should be taken with great caution.

**Figure 4:**
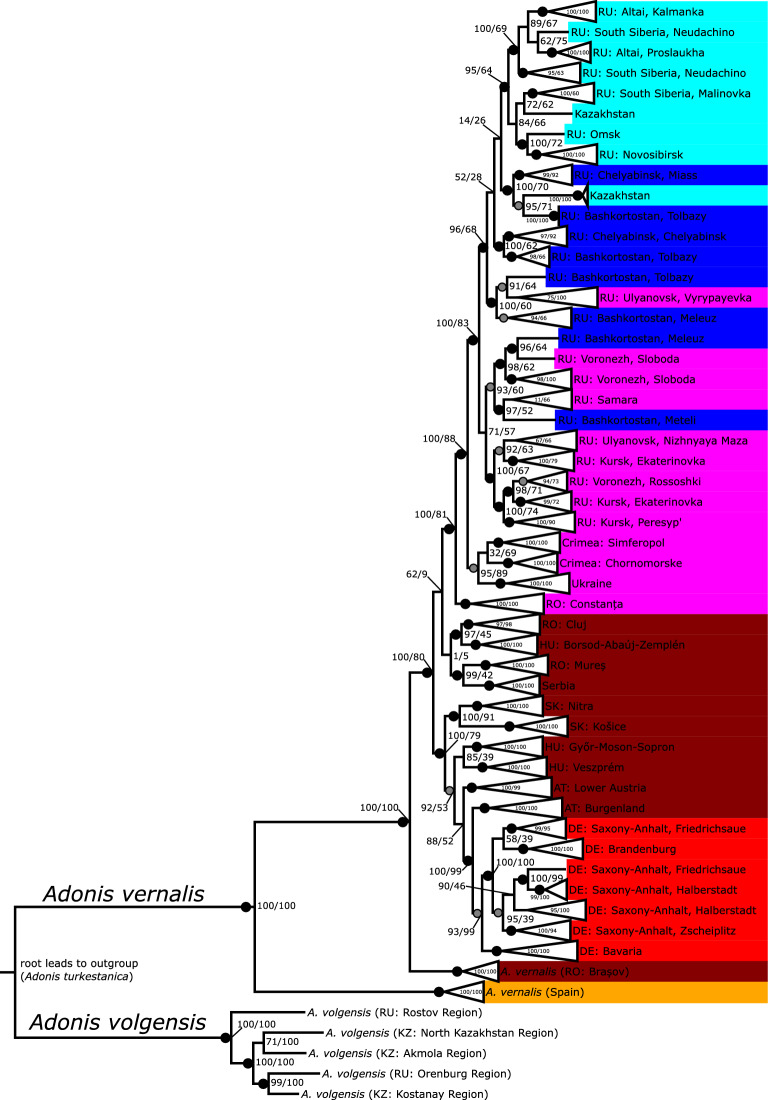
Maximum-likelihood analysis of GBS data based on unlinked SNPs from 23,242 loci (performed with IQ-TREE software). Values at nodes denote SH-like approximate likelihood ratio test and ultrafast bootstrapping support (aLRT/UFBoot) with 1,000 replicates each. Gray dots represent 90–95% and black dots 95–100% aLRT support of the nodes. *Adonis turkestanica* (truncated) was set as outgroup. Note that another species known to be closely related to *A. vernalis* (*A. villosa*; Supplementary Figs. S2 and S3 online) was not included in the GBS analysis. The colour coding refers to geographic groups in *A. vernalis* (Fig. 1, Supplementary Table S4 online): yellow = Spain, red = Germany, brown = Pannonian, pink = Pontic, blue = Southern Urals/W. Western Siberia, and turquoise = Middle/E. Western Siberia. Two-letter abbreviations are ISO alpha-2 country codes. Note that not all areas where *A. vernalis* occurs are equally well covered, e.g., populations from France, Italy and Switzerland are missing. The Burgenland population (Austria) is represented by two subpopulations.

**Figure 5:**
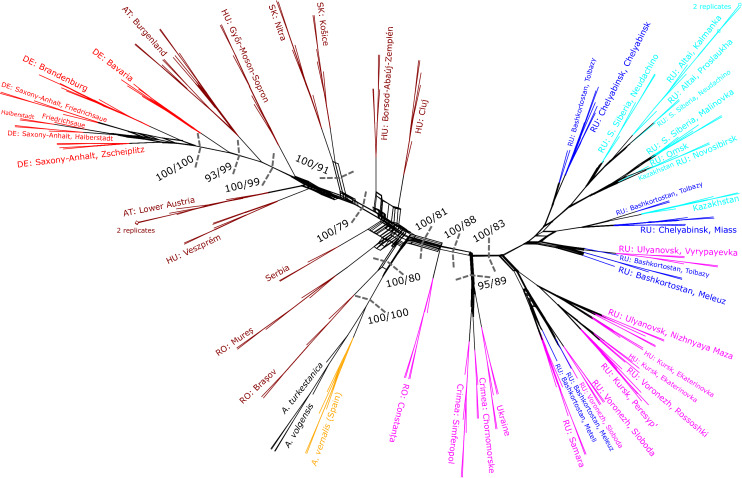
Network among *Adonis vernalis* populations plus outgroups based on GBS data. The splits used to construct the network were output from the IQ-TREE analysis, which generated the tree presented in Fig. 4. SH-like approximate likelihood ratio test and ultrafast bootstrapping (aLRT/UFBoot) support values are the same as in Fig. 4 and are only shown if both values are above 75. They are also not shown for the outgroup and for individual ingroup populations. The positions of the two replicates in the populations from Lower Austria (Austria) and Kalmanka (Russia) are indicated by diamond symbols.

A phylogenetic analysis of all *Adonis vernalis* populations plus outgroups under the ML criterion was also performed using the GBS data to find the root of the sampled *A. vernalis* populations. The result was displayed as a tree (Fig. 4) as well as a network (Fig. 5) to show any conflicting signal in the GBS data. Unfortunately, because *A. villosa* was missing in the GBS dataset, we could not resolve the conflicting relationships among *A. vernalis*, *A. villosa* and *A. volgensis* revealed by ITS and chloroplast sequences. Within *A. vernalis*, however, the Spanish population was sister to all other populations, which grouped with SH-like approximate likelihood ratio test (aLRT)/ultrafast bootstrapping (UFBoot) support equal to 100/100. Next, the Romanian population from the Brașov Depression (Inner Eastern Carpathians; Brașov County) was sister to all the remaining populations, which also grouped with very strong support (aLRT/UFBoot = 100/80). Other Pannonian populations from the Transylvanian Basin between the Eastern and Southern Carpathians in Romania (Cluj and Mureș Counties), the foot of the Bükk Mountains (Western Carpathians) in Hungary (Borsod-Abaúj-Zemplén County), and the foot of the Fruška Gora Mountain in Serbia (Vojvodina Province) grouped with very low support (aLRT/UFBoot = 1/5), essentially forming a polytomy. The remaining populations were divided into two well-supported groups. One group (aLRT/UFBoot = 100/79) comprised the Pannonian populations from Slovakia, Hungary, and Austria, as well as the German populations, whereby the Pannonian populations were paraphyletic regarding the German populations. The other group (aLRT/UFBoot = 100/81) comprised the Romanian population from Constanța County near the Black Sea coast as a sister to the populations further east, which also formed a well-supported group (aLRT/UFBoot = 100/88). East of the Constanța population, the Pontic populations from Ukraine and Crimea formed a sister group to the remaining Pontic and the Siberian populations (Russia and Kazakhstan). The network (Fig. 5) showed a west-to-east gradient among the Central European to Western Siberian *A. vernalis* populations with some signs of conflict in certain parts. Most of the conflicting signal was found next to the root (*A. turkestanica*, *A. volgensis*, and the Spanish *A. vernalis* population) in the centre of the network among the four Pannonian populations that received very little support in the tree (Cluj and Mureș Counties in Romania, Serbia, and Borsod-Abaúj-Zemplén County in Hungary), the Romanian Brașov population, and the Romanian Constanța population.

Private allelic richness (pAr) calculated from the GBS data was used as an indicator of past population sizes, and we assume that higher pAr indicates longer in situ histories of populations and/or larger population sizes in the past. The Spanish population had a similarly high pAr when compared with the group of all other populations combined (namely, 2.0 vs. 2.1 private SNPs per 10 unlinked SNPs). Comparing the geographic groups of the eastern lineage of *A. vernalis* (excluding Spain), the Pontic group had the highest value, and the middle and eastern Western Siberian group had the lowest value (Fig. 1). Population pAr values varied from 0.3 to 1.4 private SNPs per 100 unlinked SNPs (Supplementary Table S2 online). Allelic richness within populations was similar in all geographic groups (Supplementary Table S3 online).

## Discussion

The results of the phylogenetic analyses based on Sanger sequences allow us to evaluate the evolutionary history of the pheasant’s eye in the light of its phylogenetic background. The phylogenetic analyses (Supplementary Fig. S2 online) indicate that *Adonis vernalis* is closely related to *A. villosa* and *A. volgensis*. The three species differ in their distributions (with overlaps) as well as in their habitats (also with overlaps), suggesting both speciation by geographic isolation (e.g., due to Pleistocene climate fluctuations) and/or ecological speciation as possible mechanisms. In the Eastern European and Western Siberian zonal steppe area, the range of *A. volgensis* (with annual precipitation below 300 mm, formation conditions of kastanozem soil) adjoins the range of *A. vernalis* (with annual precipitation above 300 mm, formation conditions of chernozem soil) to the south^22^ (Fig. 1). *Adonis villosa*, in turn, grows in mountain steppes of Middle and Central Asia^28^. The ranges of the three species suggest a centre of diversity of the species group in Middle Asia.

The phylogenetic analyses of populations of *Adonis vernalis* based on GBS data, which are rooted with the outgroups *A. volgensis* and *A. turkestanica* (Figs. 4 and 5), show a clear distinction between the Spanish population and the Romanian Brașov population (Southeastern Europe), which is sister to the remaining populations of the eastern lineage. This suggests that recent populations of *A. vernalis* likely originated in the southern part of Europe between the Iberian Peninsula and the area west of the Black Sea, although the primary area of origin of the species group is probably Asia. Our data cannot give us an indication of whether *A. vernalis* differentiated from its closest relative in Middle Asia (where it would then have become extinct after having migrated westward) or in Eastern or Southeastern Europe, e.g., in the Black Sea region. If we consider the median ages retrieved by the molecular dating analyses, the time period between the stem group and the crown group of *A. vernalis* ranges from 1.5 to 1.6 Ma (based on ITS or chloroplast sequences, respectively) to 0.5 Ma (Supplementary Fig. S3 online, Supplementary Table S1 online). We hypothesize that the members of the lineage leading to the crown group of *A. vernalis* migrated westward to reach Spain with the expansion of open, woodless habitat during the general cooling and aridification of the Pleistocene^2,5,6,29,30^. In the southern part of Europe, close to the Mediterranean and the Black Sea, the populations at this time may have developed higher temperature and precipitation requirements and thus a higher competitive ability compared to *A. villosa* and *A. volgensis*.

The clear separation of the Spanish from the Southeastern European (Romanian) populations in *A. vernalis* has already been established by another study^31^, but it remains to be seen how other accessions from the western and central Mediterranean and the Alps (inner Alpine dry valleys) fit into the phylogenetic tree of the species^32^. The focus of our study was on the zonal Euro-Siberian steppes inhabited by the eastern lineage of *A. vernalis*. The Early-Middle Pleistocene Transition around 0.9 Ma brought a marked decrease in temperature and an increase in seasonality and aridity, with an intervening (more humid) period of (at least seasonal) reduction of open grasslands in Europe and the Mediterranean ^5,6^. This transition and the major glacial/interglacial cycles of the Middle and Late Pleistocene may have provided opportunities for population differentiation in the southern part of Europe, in the western and central Mediterranean on the one hand and in Southeastern Europe on the other hand. Particularly the Southern Carpathians and adjacent regions were recently characterized as having long-term continuity of other meadow steppe plants^33^.

Within the eastern lineage of *A. vernalis*, the analyses of population structure (Figs. 2 and 3) and the phylogenetic analyses (Figs. 4 and 5) of GBS data suggest that *A. vernalis* extended its range from the Southeastern European (Carpathian) region in two directions: It spread to the east (or northeast) and reached through the Pontic area Siberia and the region north of the Altai Mountains, and it spread to the west (or northwest) and reached northern Germany and southern Sweden. Our results thus suggest that *A. vernalis* migrated into the continental climate zone (continental forest-steppe) from the southwest (sub-Mediterranean forest-steppe in Southeastern Europe^34^).

The GBS data also give us clues about the effects of Pleistocene glaciations on populations of the pheasant’s eye. The site of the Brandenburg population in Germany was buried under the Fenno-Scandian ice shield during the ice advances of the Weichselian/Valdai Glacial corresponding to the Last Glacial Period around 115–12 ka^35^. Its private allelic richness (pAr) thus gives an indication of what value to expect (namely pAr × 100 < 0.5) if the population is postglacial in age (Supplementary Table S2 online). Southern Western Siberia (except for its westernmost part adjacent to the Ural Mountains) is the only region where all populations have similarly low pAr values. The Late Pleistocene culminated in maximum cold and aridity in the Late Valdai (= Sartan = Late Weichselian) Glaciation about 16 ka, with conditions of cold deserts in southern Western Siberia^36,37^. Postglacial (re)colonization of today’s Western Siberian forest-steppe zone^2^ could have occurred from the west (surroundings of the Ural Mountains)^38^ and/or from small-scale refugia south of the present distribution range^39^.

It may be surprising that a plant like *Adonis vernalis* should have colonized the vast area east of the Ural Mountains within a few millennia, because its dispersal ability should be limited, if one assumes only barochory (with comparatively large nutlets) and myrmecochory^20,24^ as dispersal modes. This would make the time required to colonize such a large area very high (perhaps several 100 ka^40^) even assuming a continuous habitat. Grazing, however, promotes the growth of *A. vernalis*^22^. It would therefore also be conceivable that the transport of the nutlets was by grazing ungulates (which migrated extensively in the Eurasian steppe) or specialized small mammals^1,2,39^, particularly as the nutlets have a persistent and hooked style that might get caught in fur^41,42^.

Accumulation of private alleles over time should be steady in stable populations with constant sizes. Fluctuations in population size (bottlenecks), however, should lead to a loss of (private) alleles. Private allelic richness should therefore be indicative not only of long-term in situ persistence, but also of stable population sizes over time. It seems that the proportion of open, herb-rich vegetation transitional to steppes, as it is found today in the Pontic region, was comparatively stable during the stadials and interstadials of the Weichselian/Valdai Glacial in the Black Sea region, whereas deciduous oak forests and Anatolian steppes, for example, changed more in their relative abundances^43^. It is conceivable that the vast north–south extension of the steppe and forest-steppe zone in the Pontic region of Eastern Europe allowed greater stability (with relatively easy spatial shifts) of these vegetation types during cold and warm periods (without major losses)^43,44^, while the small-scale mosaic distribution of steppe-like vegetation in the Pannonian as well as in the German (Central European) region made this more difficult^1,34,45–48^. This would explain the higher private allelic richness in the Pontic compared to the Pannonian and German region (given similar age of populations).

Finally, our data on the species history of the pheasant’s eye, together with previously published phylogeographic studies of other Euro-Siberian steppe species, contribute to our understanding of the florogenesis of the Euro-Siberian steppes. The “Pontic-Mediterranean (or sub-Mediterranean) geoelement” has been described in biogeographical literature^19^. It comprises species that are distributed both in the Pontic steppe region and in the dry (mountain) grasslands of the Mediterranean region^19^, but do not necessarily occur further east, e.g., in the Trans-Volga-Kazakh steppes. The (phylogenetic) position of the Romanian populations in the population-level analyses of GBS data (Figs. 2,3,4,5) indicates that the forest-steppe plant *A. vernalis* probably expanded its range from the sub-Mediterranean Southeastern European region west of the Black Sea into the zonal Euro-Siberian steppes (as suggested by Hoffmann^22^), providing genetic evidence that the Euro-Siberian steppe flora consists in addition to Irano-Turanian^9–17^ and Euro-Siberian^18^ also of sub-Mediterranean elements. It shows the need for a precise distinction between different steppe types with different ecological requirements to explain today’s composition of the steppe flora. The response of the steppe plants to Pleistocene climate oscillations should depend on their ecological specialization, especially with respect to temperature and humidity preferences^2^. Depending on the region, *A. vernalis* had to retreat to refugia either during the cold and continental phases of the glacials (in the Western Siberian Lowland east of the Ural Mountains) or during the forested warm and oceanic phases of the interglacials (in the Mediterranean). In Central, Southeastern and Eastern Europe west of the Ural Mountains, the species appears to have approximately maintained stable population sizes throughout the Weichselian/Valdai Glacial (with larger populations in the Pontic region of Eastern Europe as suggested by higher private allelic richness in this region). *Adonis vernalis*’ congenerics, *A. villosa* and *A. volgensis*, may have reacted differently. Since we lack phylogeographic data, we can only speculate about their Pleistocene history. However, both species occupy drier habitats and replace *A. vernalis* to the south and in the mountain steppes of Middle and Central Asia. According to Hoffmann^22^, temperature is unlikely to be a main determinant of their geographic ranges. Thus, increased aridity during the cold stages of the Pleistocene may have had less pronounced effects on these species than on *A. vernalis*. Truly drought-adapted species, like the eurythermal Irano-Turanian steppe element *Krascheninnikovia ceratoides*, in contrast, did not have to retreat during the cold periods in any region; on the contrary, this species took advantage of the cold periods to spread both south(west)ward into the Mediterranean and north(east)ward into Beringia^11,12^. This comparison shows how the different behaviour of steppe plants from different origins and with different ecological requirements determines the (heterogeneous) composition of the present steppe flora. The results of this and earlier studies are beginning to coalesce into a refined understanding of steppe florogenesis.

## Methods

### Plant material, DNA extraction and genome size measurement

Leaf material of all samples of *A. vernalis* was dried directly in the field with silica gel (Supplementary Table S4 online). Collection of plant material complied with relevant institutional, national, and international guidelines and legislation. Some other *Adonis* L. species, as well as species from the sister genus *Trollius* L., were included as outgroups and extracted from seeds when leaf material from field collections was not available (true for four samples of *A. volgensis*; see Supplementary Table S4 online). For the phylogenetic and molecular dating analyses using Sanger sequences, we included as many *Adonis* and *Trollius* species as we could collect, as well as existing sequences from GenBank. Of the total of 11 species of *Adonis* subsect. *Vernales*, which is characterized by monocyclic shoot growth^22^, we were able to include five species in addition to *A. vernalis*: *A. apennina* L., *A. turkestanica* (Korsh.) Adolf, *A. villosa* Ledeb., *A. volgensis* Steven ex DC., and *A. coerulea* Maxim. (available from GenBank). Of these, *A. turkestanica* and *A. volgensis* were also used for GBS.

We extracted genomic DNA from about 20 mg silica gel-dried leaf or seed material using the DNeasy Plant Mini Kit (Qiagen, Hilden, Germany), the NucleoSpin Plant II kit (Macherey–Nagel, Düren, Germany) or the innuPREP Plant DNA Kit using the CTAB-containing lysis solution SLS (Analytic Jena, Jena, Germany) according to the manufacturers’ instructions. The concentration of the DNA was measured by fluorescent quantification using the DeNovix dsDNA Broad Range Assay (DeNovix, Wilmington, Delaware, USA) in a DS-11 FX spectrophotometer/fluorometer (DeNovix).

To measure the genome size of three samples of *Adonis vernalis* L. by flow cytometry, we used the CyStain PI Absolute P kit (Sysmex, Görlitz, Germany) according to the manufacturer’s manual with leaf tissue of the sample and an internal standard (*Allium cepa* L. “Alice”, 2C = 34.89 pg^49^). At least 5,000 nuclei were measured in a CyFlow Space (532 nm diode laser; Sysmex) to estimate the nuclear DNA content.

### Sanger sequencing and molecular dating analysis

Three chloroplast markers (*atp*I-*atp*H intergenic spacer, *mat*K, *rpl*16 intron) and the nuclear rDNA internal transcribed spacer (ITS) region were sequenced in all available *Adonis* and *Trollius* species. For each PCR reaction, Red HS Taq Master Mix (10 μl; Biozym Scientific, Hessisch Oldendorf, Germany), forward and reverse primer (0.8 μl, 10 μM each), DNA extract (1 μl), and water (7.4 μl) was used. The PCR program for *atp*I-*atp*H with primers atpI and atpH^50^ and for *rpl*16 with primers rpL16F71 and rpL16R1516^51^ started at 95 °C for 1.5 min, followed by 35 cycles of 95 °C for 15 s, 53 °C for one minute, and 72 °C for one minute, followed by 72 °C for seven minutes and hold at 10 °C. Amplification conditions for *mat*K with primers matK-413f.-4 and matK-1227r-4^52^ were 95 °C for 1.5 min, 35 cycles of 95 °C for 30 s, 44 °C for one minute and 72° for one minute, followed by 72 °C for ten minutes and hold at 10 °C. The program for ITS with primers ITS5 and ITS4^53^ was 94 °C for two minutes, 35 cycles of 94 °C for 30 s, 50 °C for 30 s, and 72 °C for two minutes, followed by 72 °C for five minutes and 10 °C hold. Amplification products were purified by enzymatic treatment with Exonuclease I (1 μl) and FastAP Thermosensitive Alkaline Phosphatase (2 μl; Thermo Fisher Scientific, Waltham, Massachusetts, USA) at 37 °C for 15 min and at 85 °C for 15 min thereafter. Both DNA strands were sequenced. Cycle sequencing reactions consisted of BigDye Terminator v3.1 Ready Reaction Mix (1 μl; Thermo Fisher Scientific), 5 × sequencing buffer (1.5 μl), primer (1 μl, 3.5 μM; forward or reverse) and PCR product (6.5 μl). Cycling conditions after an initial denaturation at 96 °C for 1 min were 35 cycles of 96 °C for 10 s, 50 °C for 5 s and 60 °C for 4 min. Excess dye-labelled nucleotides from the sequence reaction were removed using Sephadex G-50 Fine (GE Healthcare, General Electric, Boston, Massachusetts, USA) columns prepared in MultiScreen-HV (Merck Millipore, Merck, Darmstadt, Germany) filter plates followed by a run on a 3500 Genetic Analyzer (Applied Biosystems, Thermo Fisher Scientific). The forward and reverse sequences were assembled, edited, and aligned in Geneious 6.1.8 (Biomatters, Auckland, New Zealand).

The three concatenated chloroplast loci and nuclear ITS were analysed separately. The alignments were complemented with sequences downloaded from NCBI GenBank (Supplementary Table S4 online). For maximum-parsimony analysis, the alignments were used for simple indel coding^54^ in SeqState 1.4.1^55^. The exported nexus-files with indels coded were run in PAUP*4.0a^56^ with the following settings: 10,000 bootstrap replicates, starting trees obtained via stepwise addition, random addition sequence (20 replicates), 20 trees held at each step, tree-bisection-reconnection with reconnection limit 8. For maximum-likelihood (ML) analysis, the alignments were loaded into the RAxML-NG web service maintained by Vital-IT^57^ (https://raxml-ng.vital-it.ch/#/). The three concatenated chloroplast loci each had their own GTR + FO + G evolutionary model. In ITS, ITS1 and ITS2, together with the last bases of the 5.8S rDNA (which were variable), each had their own GTR + FO + G evolutionary model (the remaining invariable bases of the rDNA were not used). The branch lengths were linked, bootstrapping with the automatic bootstopping option (bootstopping cut-off: 0.03) was requested, and the other settings were kept as default.

For molecular dating, the same alignments and partitions as in the ML analyses were defined in BEAUti v2.6.7 of the BEAST 2 package^58–60^. Each partition had its own evolutionary model (as in the ML analyses) and log normal relaxed clock model with default parameters. The trees were linked and had a Calibrated Yule model as the tree prior. We used a secondary calibration approach and normal priors for the three calibration nodes, which were fixed as monophyletic, to reflect the distribution of reported node ages^27^. The mean value of the Adonideae prior was set at 25.5 Ma, that of the *Calathodes*-*Trollius* prior at 6.6 Ma, and that of the *Megaleranthis*-*T. asiaticus* L. group prior at 2.8 Ma^27^. Two different settings were chosen for the sigma of the calibration nodes: First, sigma of all three nodes was set to 1.0, and second, sigma of the Adonideae prior was set to 5.0 and that of the other two nodes was set to 1.0 to better match the given ranges^27^. Three other groups were also fixed as monophyletic: the genus *Adonis*, the annual *Adonis* species, and the perennial *Adonis* species. The other priors had default values. The chain length was set at one billion for ITS and 200 million for the concatenated chloroplast sequences, tracelog and treelog at 5000 each. The runs for each combination of data and settings were started twice and the results were checked in Tracer v1.7.1^61^. Although the posterior and prior traces fluctuated until the end of the runs and the posterior and prior ESS were below 100, all other parameters had stable traces and ESS values well above 200. In both runs for each combination of data and settings, the median and 95% HPD interval of the age of *Adonis vernalis* in millions of years were identical after rounding to one decimal place (Supplementary Table S1 online). The runs were therefore considered stable enough. After discarding the first 10% of trees in a run as burn-in (long after reaching stationarity for all parameters except posterior and prior that continued to fluctuate throughout the runs), the maximum clade probability tree with median node heights was created in TreeAnnotator v2.6.2 of the BEAST 2 package and displayed in FigTree v1.4.3^62^.

### Genotyping-by-sequencing (GBS)

200 ng of genomic DNA were digested with the restriction enzymes *Pst*I-HF (New England Biolabs, Ipswich, Massachusetts, USA) and *Msp*I (New England Biolabs). Library preparation followed published protocols^63^. Samples were sequenced on separate lanes in two consecutive runs on a HiSeq 2500 System (Illumina, San Diego, California, USA) to achieve good genome coverage (single-end reads, fragment length approximately 100 bp). Two individuals were sequenced twice to assess the reproducibility of the method. We used ipyrad^64^ to sort, filter, de-novo assemble and align the raw data. We let ipyrad cut off the restriction site overhangs “TGCAG” and “CGG”. We set the Phred quality score to 33 and filtered reads, which had more than five low quality base calls. We applied the strict adapter filtering option to remove the common Illumina adapters. We allowed a maximum of eight indels, 20% SNPs and 50% of samples sharing heterozygous sites for a locus, up to two alleles per site, 5% uncalled bases and 5% heterozygous sites in the consensus sequence. After finding that a threshold of 85%, 90% or 95% of sequence similarity of reads to be considered reads at the same locus had a negligible effect on the topology of the tree, we decided to use a threshold of 85%. Per locus, at least 10% of all individuals (19) had to have data. Only loci with a depth of six to 10,000 and a minimum length of 35 bp were kept. We repeated the assembly with and without the outgroup species. We estimated ploidy levels from GBS data using nQuire^65^ and ploidyNGS^66^ as described in a previous publication^12^.

### Population structure and phylogenetic analysis of GBS data

To assess the population genetic structure of *A. vernalis*, we estimated individual admixture coefficients from the genotypic matrix with the LEA package^67^ in R v3.5.0^68^, with only the dataset of unlinked SNPs in the Variant Call Format (VCF) of the ingroup samples and setting the ploidy to diploid. We tested one to fifteen ancestral populations, each with 100 repetitions, and selected the correct number based on minimal cross-entropy. To display the result per population, we calculated the membership to each ancestral population per population as an average over its individuals and plotted it as a pie chart on a map. We also performed Principal Component Analysis (PCA) using the R package dartR^69^, again based on the ingroup dataset of unlinked SNPs. For LEA and PCA, the analyses were conducted twice, first with a maximum of 90% missing data per locus (37,152 loci) and second with a maximum of 10% missing data per locus (resulting in 7,795 loci with the Spanish population and 8,015 loci without the Spanish population). We filtered the vcf-file from the ipyrad output using VCFtools v0.1.13^70^ to obtain the reduced data set. The LEA and PCA results based on the larger and smaller datasets, respectively, were very similar, so we only show the result based on either the larger or smaller dataset, namely the result based on the larger dataset with a maximum of 90% missing data per locus for LEA and the result based on the smaller dataset with a maximum of 10% missing data per locus is shown for PCA.

Phylogenetic analysis including the outgroup was conducted on the dataset of unlinked SNPs of the complete sample (with a maximum of 90% missing data per locus) by applying the ML criterion using IQ-TREE v2.0.3^71^ with the GRT + G model and correction for ascertainment bias. Support of nodes was assessed using the SH-like approximate likelihood ratio test (aLRT)^72^ and ultrafast bootstrapping (UFBoot)^73^ with 1000 repetitions each. To display the splits as a network, the file with the splits output by IQ-TREE was opened in SplitsTree v4.14.5^74^.

### Private allelic richness

To calculate private allelic richness (pAr) per geographic group and (private) allelic richness (pAr and Ar) in populations within these groups, rarefaction analysis was performed in HP-Rare^75^. Only unlinked SNPs that were called in at least one individual in each population were used (8,539 of 37,152 SNPs of the ingroup dataset with a maximum of 90% missing data per locus). The rarefaction sample sizes for calculating pAr for Spain versus all other populations were one population from each group and two nucleobases from each population, and for calculating pAr for geographic groups excluding Spain, they were five populations from each group and two nucleobases from each population. The rarefaction sample size used to calculate the population Ar and pAr values were two nucleobases per population.

## Supplementary Information

Supplementary Information.

## Data Availability

The GBS raw data is available at the European Nucleotide Archive (accessions ERS10698928 to ERS10699120). Sequences generated by Sanger sequencing are available from NCBI GenBank. Please see the Supplementary Table S4 online for individual accession numbers. The alignments used for ML and BEAST analyses were made accessible through Dryad (https://doi.org/10.5061/dryad.crjdfn37h).
