# Supplementary material for: Genotyping-by-sequencing reveals range expansion of Adonis vernalis (Ranunculaceae) from Southeastern Europe into the zonal Euro-Siberian steppe
Source: Sci Rep. 2022 Nov 9;12:19074. doi: 10.1038/s41598-022-23542-w (PMC9646736; doi:10.1038/s41598-022-23542-w)
Supplement: Supplementary file 1 — Supplementary Information. [file 41598_2022_23542_MOESM1_ESM.pdf]

Supplementary Information

Genotyping-by-sequencing reveals range expansion of *Adonis vernalis* (Ranunculaceae) from Southeastern Europe into the zonal Euro-Siberian steppe

Anna Seidl[a], Karin Tremetsberger[a], Simon Pfanzelt[b,c], Lisa Lindhuber[a], Matthias Kropf[d], Barbara Neuffer[e], Frank R. Blattner[b], Gergely Király[f], Sergey V. Smirnov[g], Nikolai Friesen[h], Alexander I. Shmakov[g], Kristina Plenk[d], Oyuntsetseg Batlai[i], Herbert Hurka[e], Karl-Georg Bernhardt[a]

[a]Institute of Botany, Department of Integrative Biology and Biodiversity Research, University of Natural Resources and Life Sciences, Vienna, 1180 Vienna, Austria; [b]Experimental Taxonomy, Leibniz Institute of Plant Genetics and Crop Plant Research, 06466 Gatersleben, Germany; [c]Botanical Garden München-Nymphenburg, 80638 Munich, Germany (current affiliation); [d]Institute for Integrative Nature Conservation Research, Department of Integrative Biology and Biodiversity Research, University of Natural Resources and Life Sciences, Vienna, 1180 Vienna, Austria; [e]School of Biology/Chemistry, Osnabrück University, 49076 Osnabrück, Germany; [f]Faculty of Forestry, University of Sopron, 9400 Sopron, Hungary; [g]South-Siberian Botanical Garden, Altai State University, 656049 Barnaul, Russia; [h]Botanical Garden of the Osnabrück University, 49076 Osnabrück, Germany; [i]Department of Biology, School of Arts and Science, National University of Mongolia, 14201 Ulaanbaatar, Mongolia

Supplementary Table S1. Age estimates in millions of years obtained by molecular dating for *Adonis vernalis* and for the group comprising *A. vernalis*, *A. villosa* and *A. volgensis*. Median and 95% HPD intervals were extracted from the trees (the first run in each case is shown in Supplementary Fig. S3). The dating analyses were carried out separately for ITS sequences and for the concatenated chloroplast sequences (*atp*I-*atp*H, *mat*K, *rpl*16). Two runs were conducted for each combination of data and settings (sigma of the normal prior for the tribe Adonideae). Considering the different behavior of the runs with different values for sigma of the normal prior of the tribe Adonideae (see also Supplementary Fig. S3), we use the median of the runs with sigma = 1.0 and the 95% HPD interval of the runs with sigma = 5.0 (in bold).

Sequences	Sigma of the normal prior for the tribe Adonideae	Run	Crown group age of *A. vernalis*	Stem group age of *A. vernalis*
ITS	1.0	First	**0.49** (0.01–1.38)	**1.50** (0.41–3.04)
ITS	1.0	Second	**0.49** (0.01–1.38)	**1.50** (0.41–3.03)
ITS	5.0	First	0.51 **(0.01–1.49)**	1.59 **(0.41–3.32)**
ITS	5.0	Second	0.51 **(0.01–1.49)**	1.59 **(0.41–3.32)**
Chloroplast	1.0	First	**0.53** (0.05–1.26)	**1.59** (0.90–2.42)
Chloroplast	1.0	Second	**0.53** (0.06–1.27)	**1.58** (0.91–2.42)
Chloroplast	5.0	First	0.52 **(0.06–1.30)**	1.56 **(0.80–2.49)**
Chloroplast	5.0	Second	0.52 **(0.05–1.28)**	1.56 **(0.81–2.48)**

Sequences	Sigma of the normal prior for the tribe Adonideae	Run	Crown group age of the group comprising *A. vernalis*, *A. villosa* and *A. volgensis*	Stem group age of the group comprising *A. vernalis*, *A. villosa* and *A. volgensis*
ITS	1.0	First	**3.00** (1.28–5.26)	**5.58** (3.01–8.83)
ITS	1.0	Second	**3.00** (1.27–5.26)	**5.59** (3.01–8.82)
ITS	5.0	First	3.18 **(1.23–5.80)**	5.93 **(2.88–9.85)**
ITS	5.0	Second	3.17 **(1.22–5.79)**	5.93 **(2.83–9.81)**
Chloroplast	1.0	First	**1.59** (0.90–2.42)	**2.14** (1.22–3.22)
Chloroplast	1.0	Second	**1.58** (0.91–2.42)	**2.14** (1.23–3.21)
Chloroplast	5.0	First	1.56 **(0.80–2.49)**	2.10 **(1.11–3.32)**
Chloroplast	5.0	Second	1.56 **(0.81–2.48)**	2.10 **(1.09–3.32)**

Supplementary Table S2. Mean ± standard error (over 8,539 unlinked SNPs) of private allelic richness (pAr) × 100 in populations of *Adonis vernalis* (excluding Spain). The location of the Brandenburg population east of Berlin on the Oder River (marked with “postglacial!!”) was covered by the Fenno-Scandian ice shield during the maximum extent of the Weichselian glacial (MIS 2). This population therefore gives us an indication of what pAr value to expect (namely pAr × 100 < 0.5) if the population became established after the last glacial period. In Germany, only the Bavarian population and the southernmost (Zscheiplitz) population of the three populations from Saxony-Anhalt have higher values. The other two populations from Saxony-Anhalt (Halberstadt and Friedrichsaue), which are located somewhat further north, have a similar pAr value as the Brandenburg population. It is important to consider that populations in the never-glaciated area may also have become established postglacial from nearby refugial populations, for example, at sites that became favorable due to human activities such as clearing and grazing. This would explain their low private allelic richness. The populations in question are marked “postglacial?”.

Geographic group	Population	pAr × 100	Age
Germany	DE: Saxony-Anhalt, Halberstadt	0.38 ± 0.03	postglacial?
Germany	DE: Saxony-Anhalt, Friedrichsaue	0.41 ± 0.03	postglacial?
Germany	DE: Saxony-Anhalt, Zscheiplitz	0.91 ± 0.04	
Germany	DE: Brandenburg	0.45 ± 0.04	postglacial!!
Germany	DE: Bavaria	0.94 ± 0.04	
Pannonian	AT: Lower Austria	0.53 ± 0.04	
Pannonian	AT: Burgenland	0.53 ± 0.03	
Pannonian	SK: Nitra	0.79 ± 0.04	
Pannonian	SK: Košice	0.88 ± 0.05	
Pannonian	HU: Győr-Moson-Sopron	0.66 ± 0.05	
Pannonian	HU: Veszprém	1.02 ± 0.05	
Pannonian	HU: Borsod-Abaúj-Zemplén	0.48 ± 0.04	postglacial?
Pannonian	Serbia	1.05 ± 0.06	
Pannonian	RO: Cluj	0.55 ± 0.04	
Pannonian	RO: Mureș	0.73 ± 0.05	
Pannonian	RO: Brașov	0.60 ± 0.06	
Pontic	RO: Constanța	0.41 ± 0.03	postglacial?
Pontic	Crimea: Chornomorske	0.89 ± 0.05	
Pontic	Crimea: Simferopol	0.42 ± 0.04	postglacial?
Pontic	Ukraine	0.79 ± 0.04	
Pontic	RU: Kursk, Peresyp'	1.04 ± 0.05	
Pontic	RU: Kursk, Ekaterinovka	1.05 ± 0.05	
Pontic	RU: Voronezh, Rossoshki	1.06 ± 0.07	
Pontic	RU: Voronezh, Sloboda	0.78 ± 0.04	
Pontic	RU: Ulyanovsk, Vyrypayevka	1.29 ± 0.06	
Pontic	RU: Ulyanovsk, Nizhnyaya Maza	1.36 ± 0.06	
Pontic	RU: Samara	0.96 ± 0.04	
S. Urals/W. Western Siberia	RU: Bashkortostan, Meleuz	0.87 ± 0.04	
S. Urals/W. Western Siberia	RU: Bashkortostan, Tolbazy	0.42 ± 0.03	postglacial?
S. Urals/W. Western Siberia	RU: Bashkortostan, Meteli	0.94 ± 0.08	
S. Urals/W. Western Siberia	RU: Chelyabinsk, Miass	0.36 ± 0.03	postglacial?
S. Urals/W. Western Siberia	RU: Chelyabinsk, Chelyabinsk	0.85 ± 0.04	
Middle/E. Western Siberia	Kazakhstan	0.28 ± 0.03	postglacial?
Middle/E. Western Siberia	RU: Omsk	0.35 ± 0.05	postglacial?
Middle/E. Western Siberia	RU: South Siberia, Malinovka	0.32 ± 0.03	postglacial?
Middle/E. Western Siberia	RU: South Siberia, Neudachino	0.37 ± 0.03	postglacial?
Middle/E. Western Siberia	RU: Novosibirsk	0.26 ± 0.03	postglacial?
Middle/E. Western Siberia	RU: Altai, Proslaukha	0.33 ± 0.04	postglacial?
Middle/E. Western Siberia	RU: Altai, Kalmanka	0.36 ± 0.04	postglacial?

Supplementary Table S3. Allelic richness (Ar) in populations of *Adonis vernalis*. Population values (mean over 8,539 unlinked SNPs) were averaged for predefined geographic groups. Shown are the mean and standard deviation of population values per geographic group.

Geographic group	Number of populations	Ar
Spain	1	1.08
Germany	5	1.09 ± 0.01
Pannonian	11	1.09 ± 0.01
Pontic	11	1.10 ± 0.01
S. Urals/W. Western Siberia	5	1.09 ± 0.01
Middle/E. Western Siberia	7	1.08 ± 0.01

Supplementary Table S4. Information on samples used for Sanger sequencing (usually one individual per accession; with NCBI GenBank numbers) and for genotyping-by-sequencing (GBS; usually five individuals per accession; with EMBL-EBI ENA accession numbers). Additional taxa with complete chloroplast and internal transcribed spacer (ITS) sequence information retrieved from GenBank and used for molecular dating are also indicated with their respective numbers. Taxonomy follows Plants of the World Online (http://www.plantsoftheworldonline.org/; accessed on August 22, 2021). Vouchers are deposited at the herbarium of the University of Natural Resources and Life Sciences, Vienna (WHB) or the University of Osnabrück (OSBU). Affiliation of *Adonis vernalis* populations used for GBS to geographic groups is indicated in square brackets. *N*, number of individuals.

Adonis aestivalis L.: Austria, Lower Austria, E of Goggendorf (48.61836° N, 15.94728° E), collected by K.-G. Bernhardt et al. (May 23, 2017), WHB 68961
*atp*I-*atp*H: OL943534 *mat*K: OL943570 *rpl*16: OL943552
ITS: OL647858

Adonis amurensis Regel & Radde
MW042677.1 (complete chloroplast genome) (17)
KU570388.1, KU570389.1 (ITS) (18)

Adonis annua L.: Italy, Sicily, between Siculiana and Ribera (without coordinates), collected by K.-G. Bernhardt (April 22, 1984), OSBU 3332
*atp*I-*atp*H: OL943535 *mat*K: OL943571 *rpl*16: OL943553
ITS: OL647859

Adonis apennina L.: Mongolia, Selenge, Khonin Nuga NE of Züünkharaa/Mandal (49.06306° N, 107.27450° E), collected by O. Batlai (May 28, 2018), WHB 72473
*atp*I-*atp*H: OL943536 *mat*K: OL943572 *rpl*16: OL943554
ITS: OL647860

Adonis coerulea Maxim.
MK253469.1 (complete chloroplast genome) (19)
KU570393.1, KU570402.1 (ITS) (18)

Adonis flammea Jacq.: Hungary, Veszprém, S of Öskü (47.15550° N, 18.08019° E), collected by K.-G. Bernhardt et al. (June 1, 2018), WHB 72048
*atp*I-*atp*H: OL943537 *mat*K: OL943573 *rpl*16: OL943555
ITS: OL647861

Adonis sutchuenensis Franch.
MK569470.1 (complete chloroplast genome) (14)
KU570406.1, KU570407.1, KU570408.1 (ITS) (18)

Adonis turkestanica (Korsh.) Adolf: Tajikistan, Sughd, between Sarytag and Kaznok Pass (39.12361° N, 68.25306° E), coll. by B. Frajman & P. Schönswetter (Aug 4, 2017), WHB 71502
*atp*I-*atp*H: OL943538 *mat*K: OL943574 *rpl*16: OL943556
ITS: OL647862 GBS (*N* = 1): ERS10699029

Adonis vernalis L.: Spain, Granada, Sierra de Huétor NE of Granada (37.30139° N, 3.44556° W), collected by G. Schneeweiss & P. Schönswetter (April 25, 2005), no voucher
*atp*I-*atp*H: OL943539 *mat*K: OL943575 *rpl*16: OL943557
ITS: OL647863 GBS (*N* = 5): ERS10699078–ERS10699082 [Spain]

Adonis vernalis L.: Germany, Saxony-Anhalt, Harslebener Berge S of Halberstadt (51.83046° N, 11.07173° E), collected by F. Blattner (April 21, 2017), no voucher
*atp*I-*atp*H: same as OL943541 *mat*K: same as OL943577 *rpl*16: same as OL943559
ITS: same as OL647864 GBS (*N* = 5): ERS10698979–ERS10698983 [Germany]

Adonis vernalis L.: Germany, Saxony-Anhalt, N of Friedrichsaue (51.85763° N, 11.33289° E), collected by F. Blattner (April 15, 2017), no voucher
GBS (*N* = 5): ERS10698974–ERS10698978 [Germany]

Adonis vernalis L.: Germany, Saxony-Anhalt, Unstruthänge W of Zscheiplitz (51.21618° N, 11.72131° E), collected by F. Blattner (May 6, 2017), no voucher
GBS (*N* = 4): ERS10698984–ERS10698988 [Germany]

Adonis vernalis L.: Germany, Brandenburg, Oderberge S of Lebus (52.40544° N, 14.53353° E), collected by K.-G. Bernhardt et al. (June 15, 2017), WHB 69157
*atp*I-*atp*H: same as OL943541 *mat*K: same as OL943577 *rpl*16: same as OL943559
ITS: same as OL647864 GBS (*N* = 5): ERS10698989–ERS10698993 [Germany]

Adonis vernalis L.: Germany, Bavaria, Garchinger Heide N of Munich (48.29032° N, 11.65305° E), collected by A. Seidl (April 16, 2018), WHB 72504
GBS (*N* = 5): ERS10699089–ERS10699093 [Germany]

Adonis vernalis L.: Austria, Lower Austria, Galgenberg N of Oberstinkenbrunn (48.64889° N, 16.17000° E), collected by M. Kropf (April 4, 2010), no voucher
*atp*I-*atp*H: OL943540 *mat*K: OL943576 *rpl*16: OL943558
ITS: same as OL647864

Adonis vernalis L.: Austria, Lower Austria, Eichkogel S of Mödling (48.06292° N, 16.29056° E), collected by M. Kriechbaum (April 8, 2010), no voucher
*atp*I-*atp*H: same as OL943541 *mat*K: same as OL943577 *rpl*16: same as OL943559
ITS: same as OL647864

Adonis vernalis L.: Austria, Lower Austria, Steinberg-Spitzerberg N of Prellenkirchen (48.09514° N, 16.97203° E), collected by K.-G. Bernhardt (May 5, 2015), WHB 63392
GBS (*N* = 5): ERS10698932–ERS10698937 [Pannonian]
(ERS10698933 and ERS10698934 are replicates of the same individual.)

Adonis vernalis L.: Austria, Burgenland, between Winden am See and Jois (47.96169° N, 16.77300° E), collected by K.-G. Bernhardt (June 30, 2015), WHB 65147, WHB 68984, 68985
GBS (*N* = 10): ERS10698958–ERS10698962, ERS10698969–ERS10698973 [Pannonian]

Adonis vernalis L.: Slovakia, Nitra Region, N of Nitra (48.34945° N, 18.09278° E), collected by K. Plenk (May 24, 2017), no voucher
*atp*I-*atp*H: same as OL943541 *mat*K: same as OL943577 *rpl*16: same as OL943559
ITS: same as OL647864 GBS (*N* = 5): ERS10699068–ERS10699072 [Pannonian]

Adonis vernalis L.: Slovakia, Košice Region, NE of Kečovo (48.49328° N, 20.48883° E), collected by K. Plenk (May 25, 2017), no voucher
*atp*I-*atp*H: same as OL943541 *mat*K: same as OL943577 *rpl*16: same as OL943559
ITS: same as OL647864 GBS (*N* = 5): ERS10699073–ERS10699077 [Pannonian]

Adonis vernalis L.: Hungary, Győr-Moson-Sopron, Fertőrákos (47.72539° N, 16.64203° E), collected by G. Király (May 16, 2017), WHB 72564
*atp*I-*atp*H: same as OL943541 *mat*K: same as OL943577 *rpl*16: same as OL943559
ITS: same as OL647864 GBS (*N* = 5): ERS10699038–ERS10699042 [Pannonian]

Adonis vernalis L.: Hungary, Veszprém, Castle of Sümeg (46.98222° N, 17.28194° E), collected by K. Plenk (May 27, 2016), WHB 80603
*atp*I-*atp*H: same as OL943541 *mat*K: same as OL943577 *rpl*16: same as OL943559
ITS: same as OL647864 GBS (*N* = 5): ERS10699083–ERS10699087 [Pannonian]

Adonis vernalis L.: Hungary, Fejér, E of Belsőbáránd (47.10625° N, 18.55000° E), collected by M. Höhn (April 29, 2010), no voucher
*atp*I-*atp*H: same as OL943541 *mat*K: same as OL943577 *rpl*16: same as OL943559
ITS: same as OL647864

Adonis vernalis L.: Hungary, Pest, W of Pilisjászfalu (47.66128° N, 18.79017° E), collected by K. Bardy (May 5, 2010), WHB 80602
*atp*I-*atp*H: same as OL943541 *mat*K: same as OL943577 *rpl*16: same as OL943559
ITS: same as OL647864

Adonis vernalis L.: Hungary, Borsod-Abaúj-Zemplén, S of Szomolya (47.86292° N, 20.51653° E), collected by A. Schmotzer (May 25, 2017), WHB 72568
GBS (*N* = 5): ERS10699043–ERS10699047 [Pannonian]

Adonis vernalis L.: Serbia, Vojvodina, N of Krušedol (45.12503° N, 19.94517° E), collected by M. Kropf (May 7, 2016), no voucher
*atp*I-*atp*H: same as OL943541 *mat*K: same as OL943577 *rpl*16: same as OL943559
ITS: same as OL647864 GBS (*N* = 3): ERS10699065–ERS10699067 [Pannonian]

Adonis vernalis L.: Romania, Cluj, N of Cluj-Napoca, NNW of Vultureni (46.96814° N, 23.55361° E), collected by K.-G. Bernhardt (May 29, 2015), WHB 64548-64550
GBS (*N* = 5): ERS10698943–ERS10698947 [Pannonian]

Adonis vernalis L.: Romania, Mureș, W of Târgu Mureș, SSE of Pănet (46.53903° N, 24.47525° E), collected by K.-G. Bernhardt (May 28, 2015), WHB 64553, 64554
*atp*I-*atp*H: same as OL943541 *mat*K: same as OL943577 *rpl*16: same as OL943559
ITS: same as OL647864 GBS (*N* = 5): ERS10698953–ERS10698957 [Pannonian]

Adonis vernalis L.: Romania, Brașov, NNE of Brașov, SE of Sânpetru (45.70478° N, 25.64053° E), collected by K.-G. Bernhardt (May 27, 2015), WHB 64556
GBS (*N* = 5): ERS10698948–ERS10698952 [Pannonian]

Adonis vernalis L.: Romania, Constanța, SW of Murfatlar (44.16008° N, 28.39094° E), collected by K.-G. Bernhardt (May 20, 2015), WHB 64559
*atp*I-*atp*H: OL943541 *mat*K: OL943577 *rpl*16: OL943559
ITS: OL647864 GBS (*N* = 5): ERS10698938–ERS10698942 [Pontic]

Adonis vernalis L.: Crimea, SW of Chornomorske, 8 km NNE of Olenevka (45.44391° N, 32.58656° E), collected by P. A. Volkova & L. A. Abramova (June 6, 2015), WHB 71509
*atp*I-*atp*H: same as OL943541 *mat*K: same as OL943577 *rpl*16: same as OL943559
ITS: OL647865 GBS (*N* = 3): ERS10699035–ERS10699037 [Pontic]

Adonis vernalis L.: Crimea, SE of Simferopol, 3 km ENE of Mount Karatau (44.85453° N, 34.51514° E), collected by P. A. Volkova & L. A. Abramova (June 10, 2015), WHB 71511
*atp*I-*atp*H: same as OL943541 *mat*K: same as OL943577 *rpl*16: same as OL943559
ITS: same as OL647864 GBS (*N* = 5): ERS10699030–ERS10699034 [Pontic]

Adonis vernalis L.: Ukraine, Zaporizhzhia Region, W of Troitske (47.06439° N, 35.43555° E), collected by S. Pfanzelt et al. (July 7, 2018), WHB 74133
*atp*I-*atp*H: same as OL943541 *mat*K: same as OL943577 *rpl*16: same as OL943559
ITS: same as OL647864 GBS (*N* = 5): ERS10699109–ERS10699113 [Pontic]

Adonis vernalis L.: Russia, Kursk Region, Peresyp' SE of Oboyan', SW of Pristen' (51.09167° N, 36.48000° E), collected by A. Seidl et al. (June 14, 2018), WHB 73164
GBS (*N* = 5): ERS10699099–ERS10699103 [Pontic]

Adonis vernalis L.: Russia, Kursk Region, Ekaterinovka NE of Manturovo (51.50194° N, 37.30194° E), collected by A. Seidl et al. (June 16, 2018), WHB 73167
GBS (*N* = 5): ERS10699104–ERS10699108 [Pontic]

Adonis vernalis L.: Russia, Voronezh Region, 5 km NW of Rossoshki (51.25785° N, 38.86136° E), collected by N. Tikhomirov (May 22, 2017), WHB 72670
*atp*I-*atp*H: same as OL943541 *mat*K: same as OL943577 *rpl*16: same as OL943559
ITS: same as OL647864 GBS (*N* = 2): ERS10699053–ERS10699054 [Pontic]

Adonis vernalis L.: Russia, Voronezh Region, N of Sloboda (51.18139° N, 40.28472° E), collected by A. Seidl et al. (June 13, 2018), WHB 73172
GBS (*N* = 5): ERS10699094–ERS10699098 [Pontic]

Adonis vernalis L.: Russia, Ulyanovsk Region, 1 km NE of Vyrypayevka (54.11792° N, 47.03575° E), collected by P. A. Volkova & M. Yu. Grigoryan (July 10, 2017), WHB 72674
*atp*I-*atp*H: same as OL943541 *mat*K: same as OL943577 *rpl*16: same as OL943559
ITS: same as OL647864 GBS (*N* = 5): ERS10699055–ERS10699059 [Pontic]

Adonis vernalis L.: Russia, Ulyanovsk Region, 2 km N of Nizhnyaya Maza (52.94503° N, 47.92386° E), collected by N. Tikhomirov (May 20, 2017), WHB 72671
GBS (*N* = 5): ERS10699048–ERS10699052 [Pontic]

Adonis vernalis L.: Russia, Samara Region, 2 km NW of Klimovka (53.50139° N, 48.99197° E), collected by P. A. Volkova & M. Yu. Grigoryan (July 11, 2017), WHB 72675
GBS (*N* = 5): ERS10699060–ERS10699064 [Pontic]

Adonis vernalis L.: Russia, Republic of Bashkortostan, near Meleuz (52.92812° N, 55.86569° E), collected by S. V. Smirnov (May 18, 2017), OSBU 25539, WHB 71529
GBS (*N* = 5): ERS10699024–ERS10699028 [S. Urals/W. W. Siberia]

Adonis vernalis L.: Russia, Republic of Bashkortostan, between Tolbazy and Ufa (54.21800° N, 55.88456° E), collected by S. V. Smirnov (May 18, 2017), OSBU 25541, WHB 71524
GBS (*N* = 5): ERS10699014–ERS10699018 [S. Urals/W. W. Siberia]

Adonis vernalis L.: Russia, Republic of Bashkortostan, N of Meteli (56.03577° N, 57.95074° E), collected by P. A. Volkova & N. Tikhomirov (August 19, 2016), WHB 80617
GBS (*N* = 1): ERS10699088 [S. Urals/W. W. Siberia]

Adonis vernalis L.: Russia, Chelyabinsk Region, S of Miass (54.94453° N, 59.98342° E), collected by S. V. Smirnov (May 18, 2017), OSBU 25542
GBS (*N* = 5): ERS10698999–ERS10699003 [S. Urals/W. W. Siberia]

Adonis vernalis L.: Russia, Chelyabinsk Region, S of Chelyabinsk (54.97225° N, 61.24278° E), collected by S. V. Smirnov (May 17, 2017), OSBU 25543
GBS (*N* = 5): ERS10699004–ERS10699008 [S. Urals/W. W. Siberia]

Adonis vernalis L.: Kazakhstan, Akmola Region, N of Marinovka (52.03467° N, 69.18492° E), collected by S. V. Smirnov (May 17, 2017), OSBU 25538
*atp*I-*atp*H: same as OL943541 *mat*K: same as OL943577 *rpl*16: same as OL943559
ITS: same as OL647864 GBS (*N* = 5): ERS10699009–ERS10699013 [Middle/E. W. Siberia]

Adonis vernalis L.: Russia, Omsk Region, W of Omsk, ca 6 km NE of Moskalenki (54.98428° N, 72.05650° E), collected by S. Pfanzelt et al. (June 23, 2019), WHB 74893
GBS (*N* = 1): ERS10699115 [Middle/E. W. Siberia]

Adonis vernalis L.: Russia, South Siberia, between Novosibirsk and Omsk, Malinovka (without coordinates), collected by S. V. Smirnov (May 17, 2017), OSBU 25537, WHB 71520
*atp*I-*atp*H: same as OL943541 *mat*K: same as OL943577 *rpl*16: same as OL943559
ITS: same as OL647864 GBS (*N* = 5): ERS10698994–ERS10698998 [Middle/E. W. Siberia]

Adonis vernalis L.: Russia, South Siberia, between Barnaul and Novosibirsk, Neudachino (without coordinates), collected by S. V. Smirnov (May 17, 2017), OSBU 25536, WHB 71522
GBS (*N* = 5): ERS10699019–ERS10699023 [Middle/E. W. Siberia]

Adonis vernalis L.: Russia, Novosibirsk Region, between Verkh-Kargat and Starogornostalevo (54.69697° N, 79.19881° E), collected by S. Pfanzelt et al. (June 25, 2019), WHB 74897
GBS (*N* = 3): ERS10699116–ERS10699118 [Middle/E. W. Siberia]

Adonis vernalis L.: Russia, Altai Region, Proslaukha ca 10 km NE of Baevo (53.34014° N, 80.95722° E), collected by S. Pfanzelt et al. (June 26, 2019), WHB 74901
GBS (*N* = 2): ERS10699119–ERS10699120 [Middle/E. W. Siberia]

Adonis vernalis L.: Russia, Altai Region, ca 40 km S of Barnaul, N of Kalmanka (52.95975° N, 83.55089° E), collected by B. Neuffer et al. (May 14, 2016), OSBU 24705
*atp*I-*atp*H: same as OL943541 *mat*K: same as OL943577 *rpl*16: same as OL943559
ITS: same as OL647864 GBS (*N* = 5): ERS10698963–ERS10698968 [Middle/E. W. Siberia]
(ERS10698963 and ERS10698964 are replicates of the same individual.)

Adonis villosa Ledeb.: Kazakhstan, East Kazakhstan Region, NW of Urzhar (47.39022° N, 81.42000° E), collected by A. Seidl & K.-G. Bernhardt (July 31, 2019), WHB 76275
*atp*I-*atp*H: OL943542 *mat*K: OL943578 *rpl*16: OL943560
ITS: OL647866

Adonis volgensis Steven ex DC.: Ukraine, Kherson Region, NW of Osokorivka (47.45755° N, 33.85042° E), collected by S. Pfanzelt et al. (July 10, 2018), WHB 74132
*atp*I-*atp*H: OL943543 *mat*K: OL943579 *rpl*16: OL943561
ITS: same as OL647867

Adonis volgensis Steven ex DC.: Ukraine, Donetsk Region, SW of Nazarivka (47.31132° N, 37.07479° E), collected by S. Pfanzelt et al. (July 8, 2018), WHB 74134
*atp*I-*atp*H: OL943544 *mat*K: OL943580 *rpl*16: OL943562
ITS: same as OL647867

Adonis volgensis Steven ex DC.: Russia, Rostov Region, ca 2 km SW of Yagodinka (47.61557° N, 40.31656° E), collected by S. Pfanzelt et al. (June 15, 2018), no voucher
*atp*I-*atp*H: OL943545 *mat*K: OL943581 *rpl*16: OL943563
ITS: same as OL647867 GBS (*N* = 1): ERS10699114

Adonis volgensis Steven ex DC.: Russia, Rostov Region, Don Steppe Reserve (49.41891° N, 40.74368° E), collected by M. Kropf (May 25, 2016), no voucher
*atp*I-*atp*H: OL943546 *mat*K: OL943582 *rpl*16: OL943564
ITS: same as OL647867

Adonis volgensis Steven ex DC.: Russia, Orenburg Region, 40 km E of Orenburg (51.83006° N, 55.71411° E), collected by B. Neuffer et al. (May 19, 2017), OSBU 25528, WHB 71521
*atp*I-*atp*H: OL943547 *mat*K: OL943583 *rpl*16: OL943565
ITS: OL647867 GBS (*N* = 1): ERS10698928

Adonis volgensis Steven ex DC.: Russia, Orenburg Region, 3 km N of Kandurovka (51.54903° N, 56.70228° E), collected by B. Neuffer et al. (May 20, 2017), OSBU 25562, WHB 71519
*atp*I-*atp*H: same as OL943547 *mat*K: same as OL943583 *rpl*16: same as OL943565
ITS: same as OL647868 (extracted from a seed)

Adonis volgensis Steven ex DC.: Russia, Orenburg Region, 30 km N of Mednogorsk (51.67269° N, 57.45064° E), coll. by B. Neuffer et al. (May 21, 2017), OSBU 25611, WHB 71515
*atp*I-*atp*H: same as OL943547 *mat*K: same as OL943583 *rpl*16: same as OL943565
ITS: OL647868

Adonis volgensis Steven ex DC.: Kazakhstan, Kostanay Region, 220 km S of Kostanay (51.68892° N, 61.57342° E), coll. by B. Neuffer et al. (May 31, 2017), OSBU 25833, WHB 71517
*atp*I-*atp*H: same as OL943547 *mat*K: same as OL943583 *rpl*16: same as OL943565
ITS: same as OL647867 GBS (*N* = 1): ERS10698929 (extracted from a seed)

Adonis volgensis Steven ex DC.: Kazakhstan, North Kazakhstan Region, 30 km S of Novoishimskiy (52.94283° N, 66.62603° E), collected by B. Neuffer et al. (June 1, 2017), OSBU 25849, WHB 71518
*atp*I-*atp*H: same as OL943547 *mat*K: same as OL943583 *rpl*16: same as OL943565
ITS: same as OL647867 GBS (*N* = 1): ERS10698930 (extracted from a seed)

Adonis volgensis Steven ex DC.: Kazakhstan, Akmola Region, 10 km E of Kokshetau (53.28764° N, 69.26183° E), coll. by B. Neuffer et al. (June 2, 2017), OSBU 25874, WHB 71514
*atp*I-*atp*H: OL943548 *mat*K: OL943584 *rpl*16: OL943566
ITS: same as OL647867 GBS (*N* = 1): ERS10698931 (extracted from a seed)

Calathodes oxycarpa Sprague
MK569478.1 (complete chloroplast genome) (14)
HQ440197.1 (ITS) (20)

Megaleranthis saniculifolia Ohwi (synonym of *Trollius chosenensis* Ohwi)
FJ597983.1 (complete chloroplast genome) (21)
AY515399.1 (ITS; W. K. Lee and K. Heo, unpublished data)

Trollius asiaticus L.: Mongolia, Ulaanbaatar, N of Ulaanbaatar (48.08078° N, 106.83769° E), collected by K.-G. Bernhardt (September 8, 2017), WHB 71091, 71092
*atp*I-*atp*H: OL943549 *mat*K: OL943585 *rpl*16: OL943567
ITS: OL647869

Trollius asiaticus L.: Mongolia, Arkhangai, Duut Resort SW of Altan-Ovoo (47.31697° N, 101.65375° E), collected by K.-G. Bernhardt (September 11, 2017), WHB 71093
*atp*I-*atp*H: OL943550 *mat*K: OL943586 *rpl*16: OL943568
ITS: OL647870

Trollius europaeus L.: Austria, Lower Austria, Obersee N of Dürrenstein mountain (47.80653° N, 15.07906° E), collected by K.-G. Bernhardt (June 8, 2018), WHB 72059
*atp*I-*atp*H: OL943551 *mat*K: OL943587 *rpl*16: OL943569
ITS: OL647871

Trollius farreri Stapf
MK843818.1 (complete chloroplast genome) (22)
HQ440201.1 (ITS) (20)

Trollius ranunculoides Hemsl.
MK253447.1 (complete chloroplast genome) (19)
HQ440203.1 (ITS) (20)

Supplementary Figure S1. LEA-assigned ancestral populations (*K*) of all individuals based on the GBS data (ingroup dataset of 37,152 unlinked SNPs with a maximum of 90% missing data per locus). The most probable number of *K* is seven according to the cross-entropy value. The replicates of the two individuals that were sequenced twice, one in the population from Lower Austria (Austria), the other in a population from the Altai Region (Russia: Kalmanka), show similar proportions of belonging to genetic groups (or ancestral populations). The proportions of group membership of individuals shown here are the basis for the map representation in Fig. 2, which shows group membership of populations.

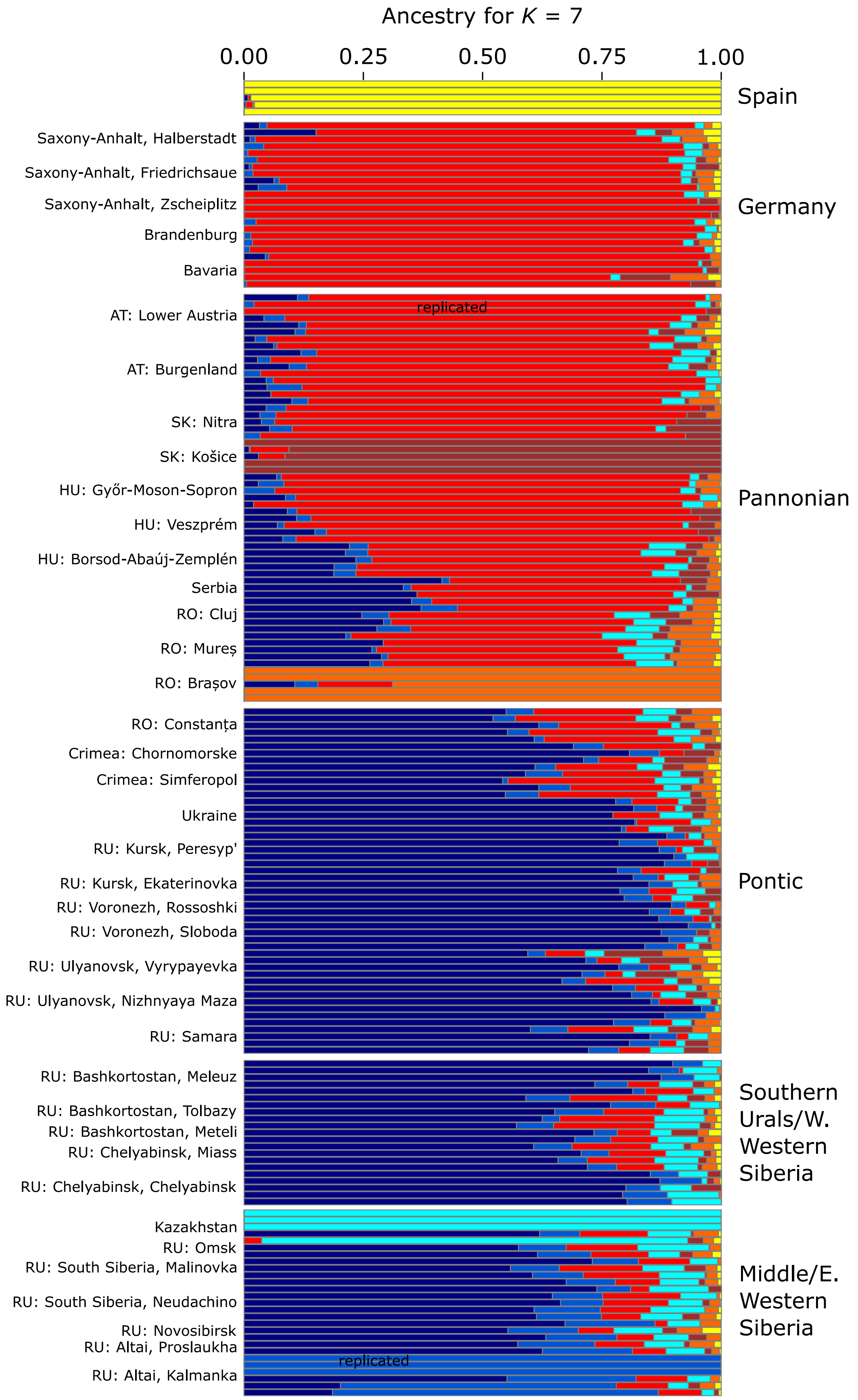

Ancestry for K = 7
0.00
0.25
0.50
0.75
1.00
Spain
Saxony-Anhalt, Halberstadt
Saxony-Anhalt, Friedrichsaue
Saxony-Anhalt, Zscheiplitz
Brandenburg
Bavaria
Germany
AT: Lower Austria
replicated
AT: Burgenland
SK: Nitra
SK: Košice
HU: Győr-Moson-Sopron
HU: Veszprém
HU: Borsod-Abaúj-Zemplén
Serbia
RO: Cluj
RO: Mureș
RO: Brașov
Pannonian
RO: Constanța
Crimea: Chornomorske
Crimea: Simferopol
Ukraine
RU: Kursk, Peresyp'
RU: Kursk, Ekaterinovka
RU: Voronezh, Rossoshki
RU: Voronezh, Sloboda
RU: Ulyanovsk, Vyrypayevka
RU: Ulyanovsk, Nizhnyaya Maza
RU: Samara
Pontic
RU: Bashkortostan, Meleuz
RU: Bashkortostan, Tolbazy
RU: Bashkortostan, Meteli
RU: Chelyabinsk, Miass
RU: Chelyabinsk, Chelyabinsk
Southern Urals/W. Western Siberia
Kazakhstan
RU: Omsk
RU: South Siberia, Malinovka
RU: South Siberia, Neudachino
RU: Novosibirsk
RU: Altai, Proslaukha
replicated
RU: Altai, Kalmanka
Middle/E. Western Siberia

Supplementary Figure S2. Results of the phylogenetic analysis of the concatenated chloroplast (left) and ITS (right) sequences. Two-letter abbreviations are ISO alpha-2 country codes. The maximum-parsimony analysis is shown at the top (bootstrap 50% majority-rule consensus tree plus other groups compatible with this tree from 10,000 bootstrap replicates). Node labels show bootstrap support values. Indels were encoded by simple indel coding. *Adonis vernalis* is represented by three multilocus chloroplast haplotypes, which differ from the other haplotypes by a 6 bp-deletion in the *atp*I-*atp*H intergenic spacer sequence. The common *A. vernalis* chloroplast haplotype is found in 20 accessions from Germany to Kazakhstan and Russia. The haplotype of the Spanish accession differs from the common haplotype by a 5 bp-insertion in the *rpl*16 intron sequence and the haplotype of the Austrian accession (Galgenberg) differs from the common haplotype by a single base substitution also in the *rpl*16 intron sequence. The common *A. volgensis* haplotype occurs in five accessions from Russia and Kazakhstan. In ITS, *A. vernalis* is also represented by three ribotypes that differ from the other ribotypes by a 1 bp-insertion and a 1 bp-substitution. As in the case of the common chloroplast haplotype, the common ribotype is also found in 20 accessions from Germany to Kazakhstan and Russia. The ribotype of the Spanish accession differs from the common ribotype in two positions by the presence of an ambiguity (namely “A” instead of “R” and “R” instead of “G” in the Spanish accession and the common ribotype). Similarly, the ribotype of the Crimean accession (Chornomorske) also differs from the common ribotype in the presence of two ambiguities (but at different positions, namely “K” instead of “G” and “R” instead of “G” in the Crimean accession and the common ribotype). The common *A. volgensis* ribotype occurs in eight accessions from Ukraine, Russia, and Kazakhstan. The maximum-likelihood analysis created with RAxML (best tree, without indel coding, with bootstrap support values) is shown at the bottom. Of the six used species of *Adonis* subsect. *Vernales* three species formed a clade in all trees: *A. vernalis*, *A. villosa* and *A. volgensis*. *Adonis turkestanica* was closely associated with this clade but formed a monophyletic group with it only in the analyses of the chloroplast sequences. Two species, however, namely *A. apennina* and *A. coerulea*, grouped with *A. sutchuenensis* Franch. or formed a polytomy with this species.

maximum parsimony

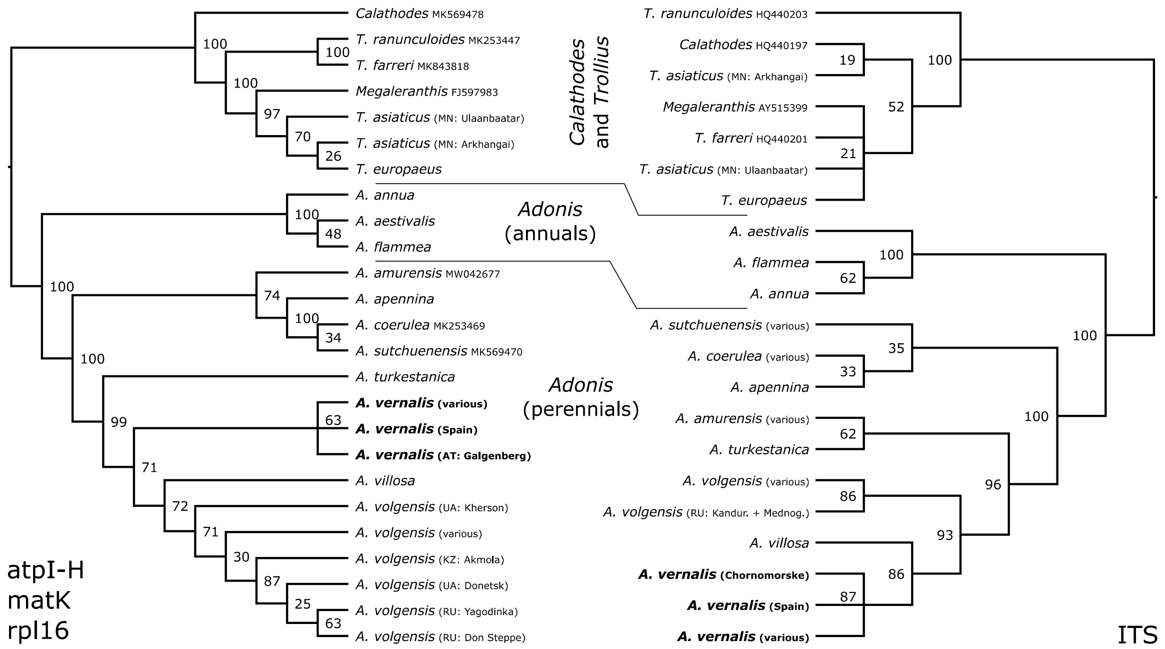

maximum likelihood

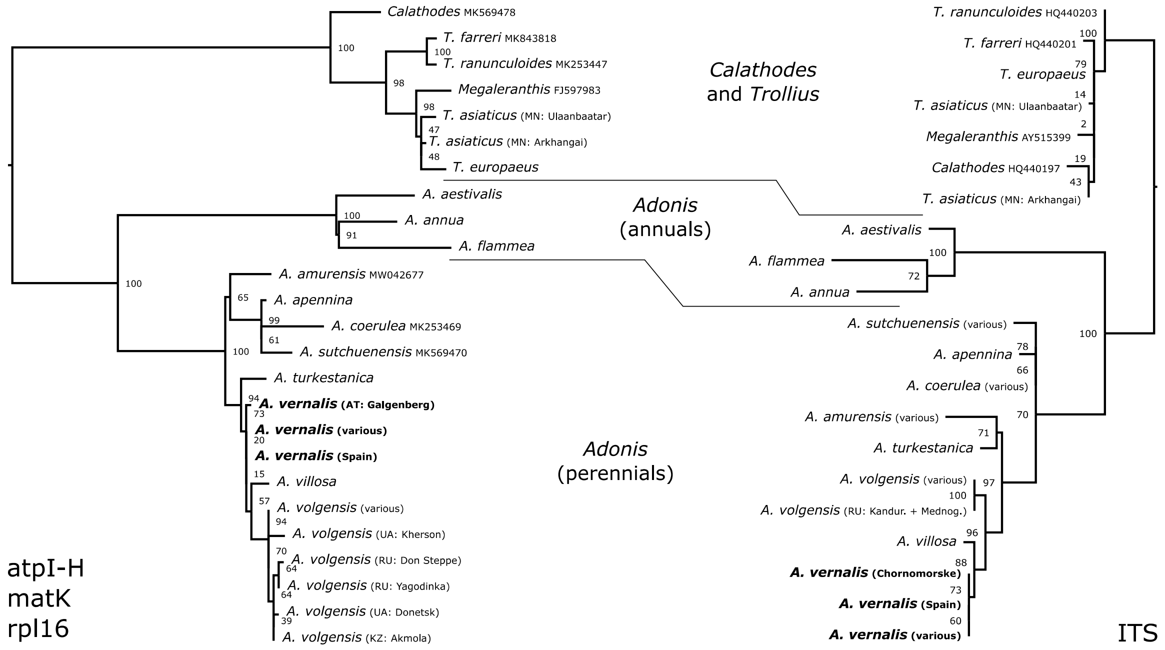

Supplementary Figure S3. Time-trees created with BEAST 2 using the ITS sequences (top) and the concatenated chloroplast sequences (*atp*I-*atp*H, *mat*K, *rpl*16; bottom). Median ages (in Ma) are displayed on the maximum clade credibility tree (node bars = 95% HPD intervals). The estimated ages of the three calibration nodes (tribe Adonideae with the mean of the normal prior set to 25.5, the clade comprising *Calathodes* and *Trollius* with the mean of the normal prior set to 6.6, and the clade comprising *Megaleranthis* and the *T. asiaticus* group with the mean of the normal prior set to 2.8) are printed in bold. For the generation of the trees on the left, the sigma of the normal prior of all three calibration nodes was set to 1.0, so that the inferred age of the tribe Adonideae is close to the set mean of the normal prior, but the 95% HPD interval does not reflect the full reported range (12.7–43.2 Ma) [27]. The trees on the right were generated with a sigma of the normal prior of the tribe Adonideae of 5.0 and otherwise unchanged settings, so the inferred age of the tribe Adonideae is shifted compared to the trees on the left, but the 95% HPD interval better reflects the reported range [27]. For each of the four combinations of data and settings, the first of two runs is shown (see also Supplementary Table S1).

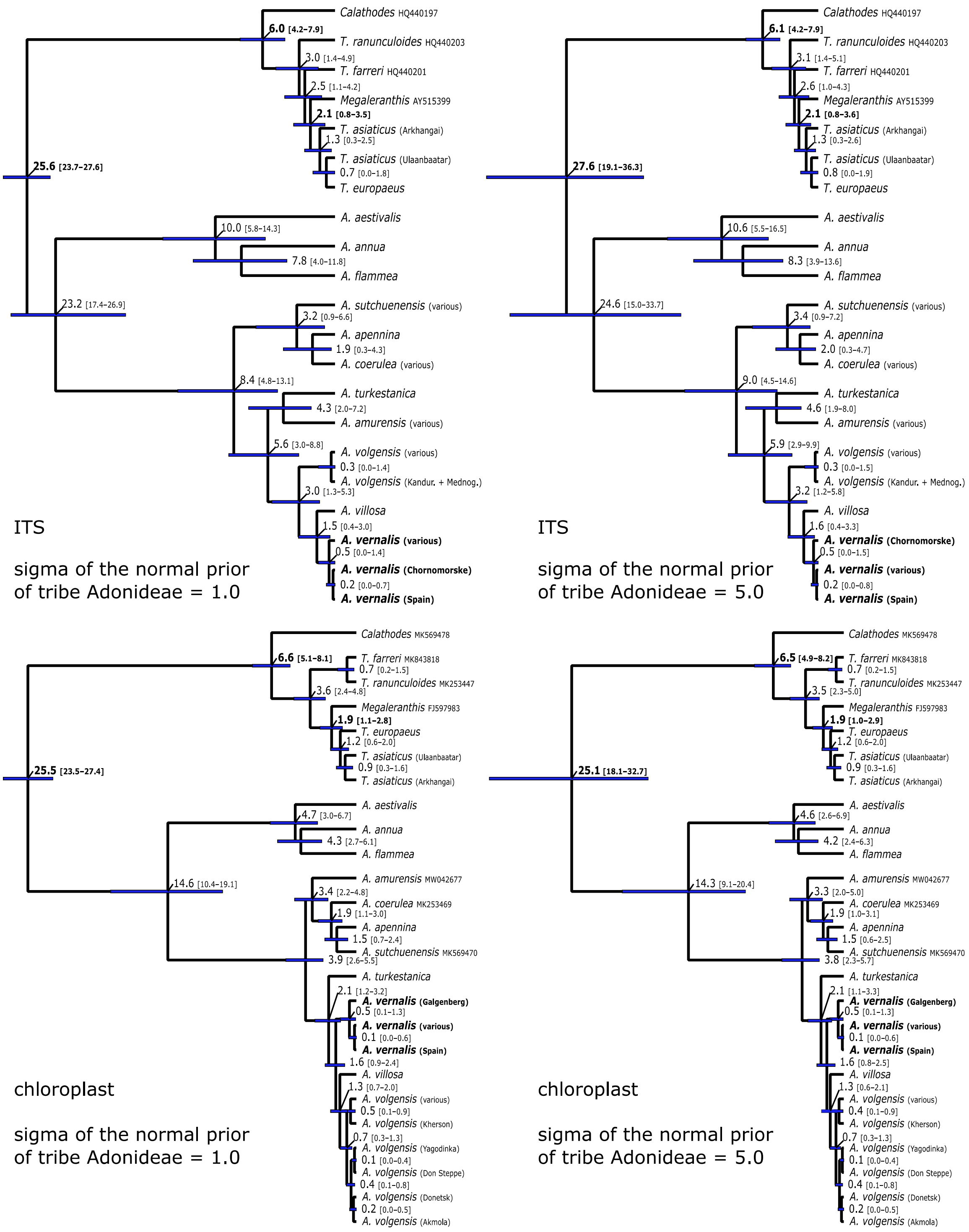
Calathodes HQ440197
6.0 [4.2–7.9]
T. ranunculoides HQ440203
3.0 [1.4–4.9]
T. farreri HQ440201
2.5 [1.1–4.2]
Megaleranthis AY515399
2.1 [0.8–3.5]
T. asiaticus (Arkhangai)
1.3 [0.3–2.5]
T. asiaticus (Ulaanbaatar)
0.7 [0.0–1.8]
T. europaeus
25.6 [23.7–27.6]
A. aestivalis
10.0 [5.8–14.3]
A. annua
7.8 [4.0–11.8]
A. flammea
23.2 [17.4–26.9]
A. sutchuenensis (various)
3.2 [0.9–6.6]
A. apennina
1.9 [0.3–4.3]
A. coerulea (various)
8.4 [4.8–13.1]
A. turkestanica
4.3 [2.0–7.2]
A. amurensis (various)
5.6 [3.0–8.8]
A. volgensis (various)
0.3 [0.0–1.4]
A. volgensis (Kandur. + Mednog.)
3.0 [1.3–5.3]
A. villosa
1.5 [0.4–3.0]
A. vernalis (various)
0.5 [0.0–1.4]
A. vernalis (Chornomorske)
0.2 [0.0–0.7]
A. vernalis (Spain)
ITS
sigma of the normal prior of tribe Adonideae = 1.0
Calathodes HQ440197
6.1 [4.2–7.9]
T. ranunculoides HQ440203
3.1 [1.4–5.1]
T. farreri HQ440201
2.6 [1.0–4.3]
Megaleranthis AY515399
2.1 [0.8–3.6]
T. asiaticus (Arkhangai)
1.3 [0.3–2.6]
T. asiaticus (Ulaanbaatar)
0.8 [0.0–1.9]
T. europaeus
27.6 [19.1–36.3]
A. aestivalis
10.6 [5.5–16.5]
A. annua
8.3 [3.9–13.6]
A. flammea
24.6 [15.0–33.7]
A. sutchuenensis (various)
3.4 [0.9–7.2]
A. apennina
2.0 [0.3–4.7]
A. coerulea (various)
9.0 [4.5–14.6]
A. turkestanica
4.6 [1.9–8.0]
A. amurensis (various)
5.9 [2.9–9.9]
A. volgensis (various)
0.3 [0.0–1.5]
A. volgensis (Kandur. + Mednog.)
3.2 [1.2–5.8]
A. villosa
1.6 [0.4–3.3]
A. vernalis (Chornomorske)
0.5 [0.0–1.5]
A. vernalis (various)
0.2 [0.0–0.8]
A. vernalis (Spain)
ITS
sigma of the normal prior of tribe Adonideae = 5.0
Calathodes MK569478
6.6 [5.1–8.1]
T. farreri MK843818
0.7 [0.2–1.5]
T. ranunculoides MK253447
3.6 [2.4–4.8]
Megaleranthis FJ597983
1.9 [1.1–2.8]
T. europaeus
1.2 [0.6–2.0]
T. asiaticus (Ulaanbaatar)
0.9 [0.3–1.6]
T. asiaticus (Arkhangai)
25.5 [23.5–27.4]
A. aestivalis
4.7 [3.0–6.7]
A. annua
4.3 [2.7–6.1]
A. flammea
14.6 [10.4–19.1]
A. amurensis MW042677
3.4 [2.2–4.8]
A. coerulea MK253469
1.9 [1.1–3.0]
A. apennina
1.5 [0.7–2.4]
A. sutchuenensis MK569470
3.9 [2.6–5.5]
A. turkestanica
2.1 [1.2–3.2]
A. vernalis (Galgenberg)
0.5 [0.1–1.3]
A. vernalis (various)
0.1 [0.0–0.6]
A. vernalis (Spain)
1.6 [0.9–2.4]
A. villosa
1.3 [0.7–2.0]
A. volgensis (various)
0.5 [0.1–0.9]
A. volgensis (Kherson)
0.7 [0.3–1.3]
A. volgensis (Yagodinka)
0.1 [0.0–0.4]
A. volgensis (Don Steppe)
0.4 [0.1–0.8]
A. volgensis (Donetsk)
0.2 [0.0–0.5]
A. volgensis (Akmola)
chloroplast
sigma of the normal prior of tribe Adonideae = 1.0
Calathodes MK569478
6.5 [4.9–8.2]
T. farreri MK843818
0.7 [0.2–1.5]
T. ranunculoides MK253447
3.5 [2.3–5.0]
Megaleranthis FJ597983
1.9 [1.0–2.9]
T. europaeus
1.2 [0.6–2.0]
T. asiaticus (Ulaanbaatar)
0.9 [0.3–1.6]
T. asiaticus (Arkhangai)
25.1 [18.1–32.7]
A. aestivalis
4.6 [2.6–6.9]
A. annua
4.2 [2.4–6.3]
A. flammea
14.3 [9.1–20.4]
A. amurensis MW042677
3.3 [2.0–5.0]
A. coerulea MK253469
1.9 [1.0–3.1]
A. apennina
1.5 [0.6–2.5]
A. sutchuenensis MK569470
3.8 [2.3–5.7]
A. turkestanica
2.1 [1.1–3.3]
A. vernalis (Galgenberg)
0.5 [0.1–1.3]
A. vernalis (various)
0.1 [0.0–0.6]
A. vernalis (Spain)
1.6 [0.8–2.5]
A. villosa
1.3 [0.6–2.1]
A. volgensis (various)
0.4 [0.1–0.9]
A. volgensis (Kherson)
0.7 [0.3–1.3]
A. volgensis (Yagodinka)
0.1 [0.0–0.4]
A. volgensis (Don Steppe)
0.4 [0.1–0.8]
A. volgensis (Donetsk)
0.2 [0.0–0.5]
A. volgensis (Akmola)
chloroplast
sigma of the normal prior of tribe Adonideae = 5.0
